# Chemical Compositions and Mosquito Larvicidal Activities of Essential Oils from *Piper* Species Growing Wild in Central Vietnam

**DOI:** 10.3390/molecules24213871

**Published:** 2019-10-27

**Authors:** Le Thi Huong, Nguyen Huy Hung, Do Ngoc Dai, Thieu Anh Tai, Vu Thi Hien, Prabodh Satyal, William N. Setzer

**Affiliations:** 1 School of Natural Science Education, Vinh University, 182 Le Duan, Vinh City 43000, Vietnam; lehuong223@gmail.com; 2 Center for Advanced Chemistry, Institute of Research and Development, Duy Tan University, 03 Quang Trung, Da Nang 50000, Vietnam; 3 Faculty of Agriculture, Forestry and Fishery, Nghe An Economics University, Vinh City 43000, Vietnam; daidn23@gmail.com; 4 Department of Pharmacy, Duy Tan University, 03-Quang Trung, Da Nang 50000, Vietnam; anhtai0808qn@gmail.com; 5 Faculty of Hydrometerology, Ho Chi Minh City University of Natural Resources and Environment, Ho Chi Minh City 70000, Vietnam; hiensphoa@gmail.com; 6 Aromatic Plant Research Center, 230 N 1200 E, Suite 100, Lehi, UT 84043, USA; psatyal@aromaticplant.org; 7 Department of Chemistry, University of Alabama in Huntsville, Huntsville, AL 35899, USA

**Keywords:** piperaceae, mosquito-borne diseases, natural pest control

## Abstract

Mosquitoes are the deadliest animals on earth and are the vectors of several neglected tropical diseases. Recently, essential oils have emerged as potential renewable, cost-effective, and environmentally benign alternatives to synthetic pesticides for control of mosquitoes. In this work, thirteen species of *Piper* were collected from different areas of central Vietnam. The essential oils were obtained by hydrodistillation and analyzed by gas chromatography–mass spectrometry. The essential oils were screened for mosquito larvicidal activity against *Aedes aegypti*. Four of the *Piper* essential oils showed outstanding larvicidal activity against *Ae. aegypti*, namely *P. caninum*, *P. longum*, *P. montium*, and *P. mutabile*, with LC_50_ and LC_90_ values less than 10 µg/mL. Multivariate analysis has correlated concentrations of β-caryophyllene, β-bisabolene, α-pinene, and β-pinene with mosquito larvicidal activity.

## 1. Introduction

The genus *Piper* is the largest in the Piperaceae and is made up of around 1050 species, many of which are important in traditional medicine and as culinary spices [1]. For example, *P. nigrum* (black pepper) fruit is the most consumed spice in the world, *P. longum* (long pepper) fruiting spikes are used similarly, *P. methysticum* (kava) root is used to produce an intoxicating herbal medicine, *P. betle* (betel) leaves are used to wrap areca (*Areca catechu*) nuts or tobacco (*Nicotiana* spp.) for chewing, and *P. cubeba* (cubeb) fruits are used to produce an essential oil [2]. The genus has shown high ethnobotanical utility worldwide and is of interest to a variety of fields and industries, including pharmaceutical botany, traditional medicine, aromatic industries, foods, and landscaping [3,4]. In Vietnam, there are about 45 species of *Piper* [5].

Mosquitoes have been and continue to be the deadliest animals on earth and are responsible for several of the world’s deadliest diseases, including malaria, yellow fever, dengue, filariasis, and many others [6]. Mosquito-borne infectious diseases have also been a constant problem in Vietnam. Dengue fever and dengue hemorrhagic fever are especially problematic and chikungunya fever is an emerging threat in the country [7,8]. *Aedes aegypti* (L.), the yellow fever mosquito, is an important vector of viral diseases including yellow fever [9], dengue [10], chikungunya [11], Zika [12], as well as others. *Aedes albopictus* (Skuse) (Diptera: Culicidae), the Asian tiger mosquito, is also an important vector of several viral pathogens, including dengue fever virus [13], yellow fever virus [14], chikungunya fever virus [15], and possibly Zika virus [16]. *Culex quinquefasciatus* Say (Diptera: Culicidae), the southern house mosquito, is a vector of lymphatic filariasis [17] as well as several arboviruses such as West Nile virus and St. Louis encephalitis virus [18], and possibly Zika virus [19].

There is a need for complementary vector control methods for controlling the spread of mosquito-borne diseases; resistance to synthetic insecticides is increasing worldwide [20,21,22,23,24], and the deleterious impacts of synthetic insecticides to the environment have been a major problem for many years [25,26]. Essential oils may provide environmentally safe and renewable alternatives to synthetic insecticides for mosquito control [27,28,29,30,31]. In this work, we present the essential oil compositions and mosquito larvicidal activities of several species of *Piper* growing wild in central Vietnam [32,33]:

*Piper arboricola* C. DC. (syn. *Piper kadsura* (Choisy) Ohwi, *Piper futokadsura* Siebold, *Piper subglaucescens* C. DC., local Vietnamese name Tiêu thượng mộc, is an understory liana native to China and Taiwan. In Vietnam, *P. arboricola* is found in Hà Tĩnh (Vũ Quang National Park), Thừa Thiên Huế Pro (Nam Đông District), and Lâm Đồng Province (Đà Lạt City: Đatanla).

*Piper bavinum* C. DC., local Vietnamese name Tiêu ba vì, is an understory climber found in China. In Vietnam, *P. bavinum* is found in Hà Nội City (Ba vì: Cốc Village) and Kon Tum Province.

*Piper cambodianum* P. Fourn. is found in Vietnam in Nghệ An, Đà Nẵng, and Ninh Thuận Provinces.

*Piper caninum* C. DC., Vietnamese name Tiêu chó, ranges from India, through Thailand, Malaysia, and Indonesia. In Vietnam, *P. caninum* is found in Kon Tum Province (Đác Tung).

*Piper longum* L., Vietnamese names Tiêu lá tím, Tất bạt, Tiêu dài, Tiêu lốt, and Trầu không dại, is an understory liana, which is distributed throughout India, China, Malaysia, Nepal, and Sri Lanka, as well as Vietnam.

*Piper mekongense* C. DC. (syn. *Piper polysyphonum* C. DC.), Vietnamese name Tiêu cửu long, is an understory shrub found in Laos, Cambodia, as well as Kon Tum province, Vietnam.

*Piper montium* C. DC., Vietnamese name Tiêu núi, is an understory climber. In Vietnam, the plant is found in Ninh Bình and Kon Tum provinces.

*Piper mutabile* C. DC., Vietnamese names Tiêu biến thể, Trầu biến thể, and Mâu linh, is an understory climber that typically grows on slopes in thickets along streams at elevations around 400–600 m. It is found in China as well as Quảng Ninh, Ninh Bình, Thanh Hóa, Nghệ An, and Kon Tum provinces of Vietnam.

*Piper nigrum* L., Vietnamese names Hồ tiêu and Tiêu, is a widespread liana, believed to have originated in India and Indonesia, but now cultivated throughout the tropics, including Vietnam provinces of Quảng Trị, Quảng Nam, Kon Tum, Đắc Lắc, Lâm Đồng, An Giang (Châu Đốc), and Kiên Giang (Phú Quốc).

*Piper politifolium* C. DC., Vietnamese name Tiêu lá láng, is native to the Neotropics, but introduced to Vietnam and found in the provinces Kon Tum (Đác Giây, Đác Môn), Gia Lai (An Khê, Kon Hà Nừng), Lâm Đồng (Bảo Lộc), and Đồng Nai.

*Piper rubrum* C. DC., Vietnamese name Tiêu đỏ, is an understory climber found in China as well as Vietnam, Phú Thọ (Tam Thanh), Ninh Bình, Quảng Nam.

*Piper sarmentosum* Roxb., Vietnamese names Lốt and Trầu già, is an herb growing in forests and wet places near villages, from sea level to 1000 m. The plant ranges from India and China through Thailand and Malaysia. In Vietnam, *P. sarmentosum* is found in the provinces of Nghệ An and Đà Nẵng.

*Piper umbellatum* L. (syn. *Piper peltatum* Ruiz & Pav., *Pothomorphe peltata* (L.) Miq., *Pothomorphe umbellata* (L.) Miq.) is an understory herb, native to the Neotropics, but introduced to Vietnam.

## 2. Results and Discussion

### 2.1. Plant Collection and Essential Oils

The plants materials (leaves and/or stems) from 13 *Piper* species were collected from various sites in Vietnam. The collection sites, plant materials, and essential oil yields for the *Piper* species are summarized in Table 1.

### 2.2. Essential Oil Compositions

The Vietnamese *Piper* species were analyzed by gas chromatography-mass spectrometry (GC-MS). A total of 272 compounds were identified in the *Piper* essential oils, which are compiled in Table 2. β-Caryophyllene was relatively abundant in all of the *Piper* essential oils and ranged from 4.0% (*P. mekongense*) to 44.8% (*P. umbellatum*). Also found in all of the essential oils were α-pinene (0.2–19.3%), β-pinene (0.1–26.9%), limonene (0.4–23.2%), α-copaene (0.2–13.7%), β-elemene (0.3–3.3%), and α-humulene (0.5–6.1%). β-Bisabolene was particularly abundant in *P. sarmentosum* (40.3%), *P. montium* (22.1%), and *P. mutabile* (12.9%), while decanal was an abundant component of *P. rubrum* stem essential oil (31.6%), and *P. nigrum* was rich in δ-elemene (20.4%).

Interestingly, many *Piper* essential oils are rich in phenylpropanoids [2], particularly *Pipers* from the Neotropics [34,35]. In this current study, however, phenylpropanoids were generally found to be lacking, with the exception of *P. politifolium*, which had asaricin (18.7%) and safrole (2.7%). As far as we are aware, there have been no previous studies on the essential oils of *P. arboricola*, *P. cambodianum*, *P. caninum*, *P. mekongense*, *P. montium*, *P. mutabile*, *P. politifolium*, or *P. rubrum*.

The essential oil from the leaves and stems of *P. bavinum*, collected in Hainan, China, was found to be rich in spathulenol (8.0%), caryophyllene oxide (7.8%), propiopiperone (7.8%), and 2,4,6-trimethylbenzenepropanol (26.9%) [36]. The identification of 2,4,6-trimethylbenzenepropanol is doubtful, however; the identification was based only on the mass spectrum (retention indices not reported) and the compound is not found in the *Dictionary of Natural Products* [37]. In contrast, the essential oil of *P. bavinum* from Huong Son, Ha Tinh Province, Vietnam, was composed largely of bicyclogermacrene (10.6%), globulol (5.7%), viridiflorene (5.1%), α-pinene (4.4%), viridiflorol (3.5%), terpinen-4-ol (3.2%), and α-gurjunene (3.0%) [38]. The major components in *P. bavinum* essential oil in this study were bicyclogermacrene (8.9%), cyclocolorenone (7.9%), β-caryophyllene (6.2%), α-humulene (6.1%), γ-curcumene (5.8%), as well as α-pinene (3.3%), α-gurjunene (1.1%), and viridiflorol (1.0%). Thus, there are qualitative similarities between the two Vietnamese *P. bavinum* samples.

The phytochemistry of *P. longum* has been reviewed [2,39,40]. The leaf essential oil of *P. longum* from the Western Ghats region of Kerala, India, had elemol (22.5%), β-caryophyllene (16.8%), and α-humulene (5.8%) as the major components, while the stem essential oil was dominated by β-pinene (34.8%), α-pinene (14.0%), limonene (10.3%), and β-caryophyllene (9.3%) [41]. A report on the leaf essential oil of *P. longum* from Nghệ An Province, Vietnam showed the major components to be fonenol (40.5%) and elemol (8.2%) [42], and is, therefore, remarkably different from the *P. longum* essential oil in this present study, which was composed largely of α-pinene (5.1%), β-pinene (10.6%), β-caryophyllene (7.9%), (*E*)-nerolidol (8.1%), and two unidentified sesquiterpenoids (5.7% and 11.9%).

The major components in the essential oil from the leaves and stems of *P. nigrum* from Vietnam in this study were δ-elemene (20.4%), β-caryophyllene (7.7%), and β-selinene (5.1%). There have been numerous studies on the essential oils of *P. nigrum*, mostly on the fruit essential oils, but the leaves and stems have also been studied [2]. There are large variations in the leaf essential oil compositions of *P. nigrum* from India depending on the geographical location [43] and the particular cultivar [44]. Indian *P. nigrum* leaf oils are generally dominated by (*E*)-nerolidol, β-caryophyllene, germacrene D, and elemol, but show wide variation in concentrations [43,44]. In contrast, the leaf essential oil of *P. nigrum* “Bragantina”, cultivated in Belém, Pará State, Brazil, showed cubenol (14.6–21.5%), δ-cadinene (2.7–5.5%), bicyclogermacrene (6.4–8.2%), β-selinene (4.1–6.2%), α-copaene (3.5–5.2%), and α-gurjunene (3.5–5.1%) as the major components [45], while the essential oil from Hainan, China, was rich in β-caryophyllene (13.8%), spathulenol (6.2%), and caryophyllene oxide (6.0%) [36].

The phytochemistry of *P. sarmentosum* has also been reviewed [2,46]. The essential oils of *P. sarmentosum* have shown great variation, depending on geographical location. The essential oil from the leaves and stems of *P. sarmentosum* collected in Guangzhou, China, had asaricin (54.5%) and palmitic acid (8.2%) as major components [36]. On the other hand, the leaf essential oil of *P. sarmentosum* from Kuching, Sarawak, Malaysia, was composed largely of spathulenol (21.0%), myristicin (18.8%), β-caryophyllene (18.2%), and (*E*,*E*)-farnesol (10.5%) [47]. Furthermore, the essential oil from leaves and stems of *P. sarmentosum* from China (location not provided) was dominated by myristicin (65.2%) and β-caryophyllene (13.9%) [48]. The leaf essential oil of *P. sarmentosum* from Bạch Mã National Park, central Vietnam, was made up largely of benzenoids, benzyl benzoate (49.1%), benzyl alcohol (17.9%), benzyl salicylate (10.0%), and (2*E*)-butenylbenzene [49]. In complete contrast to these previous studies, the essential oil composition of *P. sarmentosum* essential oil from Da Nang, Vietnam, in this study, was composed of β-bisabolene (40.3%), β-pinene (5.5%), β-caryophyllene (5.5%), and caryophyllene oxide (5.1%) as the major components; neither asaricin, palmitic acid, myristicin, nor benzyl derivatives were observed in the present Vietnamese sample.

**Figure 1:**
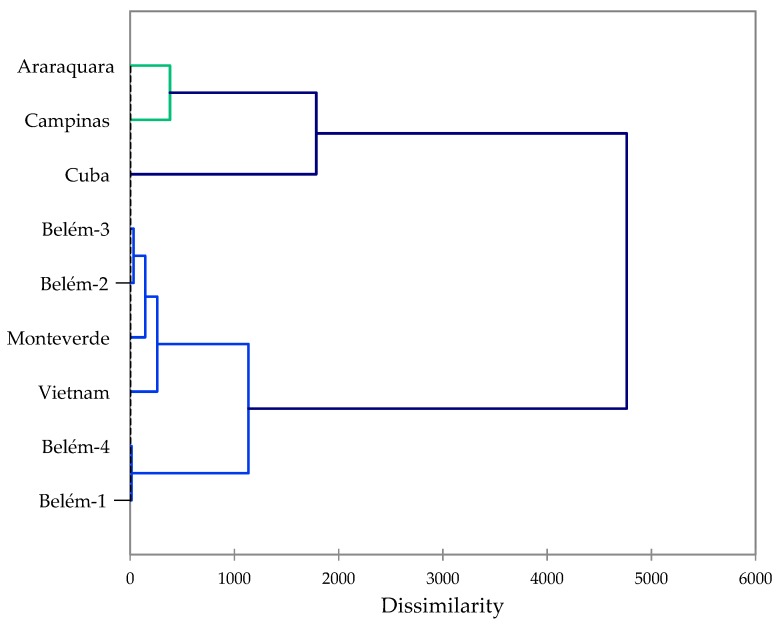
Agglomerative hierarchical cluster analysis based on the major components in *Piper umbellatum* essential oils.

*P. umbellatum*, native to the Neotropics, has been investigated from several geographical locations, including Belém, Pará State, Brazil [50]; Campinas, São Paulo State, Brazil [51]; Araraquara, São Paulo State, Brazil [52]; Monteverde, Costa Rica [53]; and Topes de Collantes, Cuba [54]. The major components of *P. umbellatum* essential oils are summarized in Table 3. A cluster analysis based on the concentrations of the major components reveals two clearly defined groups. The *P. umbellatum* essential oil fits into a cluster along with essential oils from Monteverde, Costa Rica, and Belém, Brazil (Figure 1).

### 2.3. Larvicidal Activities

Several of the *Piper* essential oils, depending on essential oil availability and availability of mosquito larvae, were screened for larvicidal activity against *Aedes aegypti*, and, when available, *Aedes albopictus* and *Culex quinquefasciatus*. The larvicidal activities are summarized in Table 4. According to a review by Dias and co-authors, plant essential oils are considered to have *Ae. aegypti* larvicidal activity if the LC_50_ values are less than 100 µg/mL [55]. Based on this criterion, all of the *Piper* essential oils in this investigation are active against larvae of this mosquito. Furthermore, four of the *Piper* essential oils showed outstanding larvicidal activity against *Ae. aegypti*, namely *P. caninum*, *P. longum*, *P. montium*, and *P. mutabile*, which had LC_50_ and LC_90_ values less than 10 µg/mL. In addition, *P. nigrum* essential oil had LC_50_ < 10 µg/mL and LC_90_ < 20 µg/mL. Although *P. cambodianum* had LC_50_ less than 10 µg/mL, the LC_90_ values were greater than 30 µg/mL.

**Figure 2:**
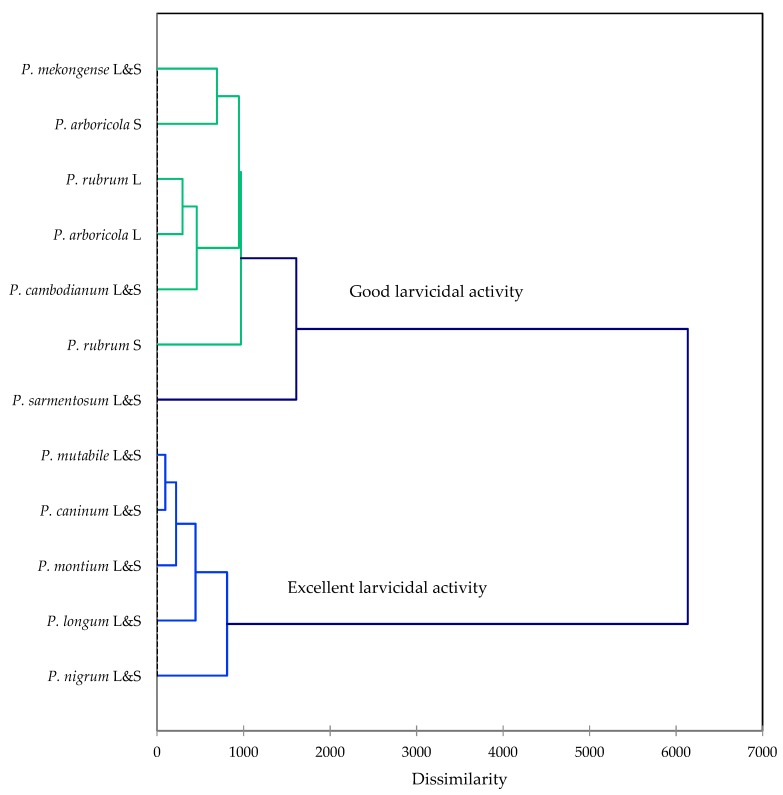
Agglomerative hierarchical cluster analysis based on the major components of the *Piper* essential oils from central Vietnam along with larvicidal activities (LC_50_ and LC_90_) against *Aedes aegypti*.

In order to provide insight into the components responsible for the larvicidal activity, a multivariate analysis, agglomerative hierarchical cluster (AHC), and principal component analysis (PCA) were carried out to correlate composition with activity. The cluster analysis revealed two clearly defined clusters, one cluster with excellent larvicidal activity (*P. mutabile*, *P. caninum*, *P. montium*, *P. longum*, and *P. nigrum*) and another cluster of *Piper* essential oils with good larvicidal activity (Figure 2).

**Figure 3:**
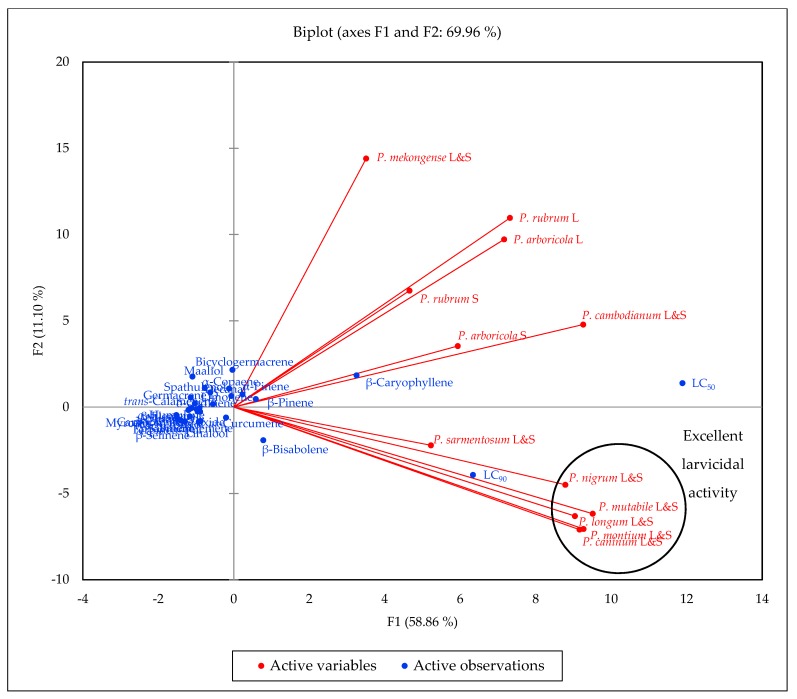
Principal component biplot of PC1 and PC2 scores and loadings indicating the correlation of chemical components of *Piper* essential oils from central Vietnam and *Aedes aegypti* larvicidal activity.

The principal component analysis indicates a positive correlation between *Ae. aegypti* larvicidal activity (LC_50_ and LC_90_) and concentrations of β-caryophyllene, β-bisabolene, α-pinene, and β-pinene (Figure 3). In addition, *P. caninum*, *P. montium*, and *P. mutabile* essential oils had relatively high concentrations of *ar*-curcumene; *P. caninum*, *P. longum, P. montium*, and *P. mutabile* had relatively high concentrations of linalool; and *P. nigrum* essential oil was rich in δ-elemene.

Several *Piper* essential oils had previously demonstrated larvicidal activity against *Ae. aegypti* [55,56,57]. The fruit essential oil of *P. aduncum* from northeastern Brazil was found to be rich in both β-pinene (32.7%) and β-caryophyllene (17.1%) and showed larvicidal activity with LC_50_ of 30.2 µg/mL [58]. Similarly, *P. nigrum* fruit essential oil with β-caryophyllene (24.2%) and β-pinene (5.4%) had LC_50_ of 75.8 µg/mL [58]. The phenylpropanoid-rich essential oils of *P. permucronatum* and *P. hostmanianum* showed larvicidal activity against *Ae. aegypti* of 36 and 54 µg/mL, respectively [56]. Furthermore, *P. sarmentosum* essential oil from Thailand, dominated by croweacin (71.0%) and β-caryophyllene (7.4%), showed very good mosquito larvicidal activity against *Anopheles cracens* [59]. In addition, α-pinene, β-pinene [60], linalool [61], and β-caryophyllene [62] had previously shown *Ae. aegypti* larvicidal activities with LC_50_ of 15.4, 12.1, 38.6, and 88.3 µg/mL, respectively.

## 3. Materials and Methods

### 3.1. Plant Material

*Piper* plant material (leaves and stems) was collected from several different locations in central Vietnam (Table 1). The plants were identified by Dr. Do Ngoc Dai, and voucher specimens (see Table 1) have been deposited in the Pedagogical Institute of Science, Vinh University. The fresh plant materials (2.0 kg each) were shredded and hydrodistilled for 4 h using a Clevenger type apparatus (Witeg Labortechnik, Wertheim, Germany). Essential oil yields are summarized in Table 1.

### 3.2. Gas Chromatographic-Mass Spectral Analysis

Each of the *Piper* essential oils was analyzed by gas chromatography–mass spectrometry (GC-MS) as previously described [63]: Shimadzu GCMS-QP2010 Ultra (Shimadzu Scientific Instruments, Columbia, MD, USA), electron impact (EI) mode (electron energy = 70 eV), scan range = 40–400 atomic mass units, scan rate = 3.0 scans/s, and GC–MS solution software. The GC column was a ZB-5 fused silica capillary column (30 m length × 0.25 mm internal diameter) with a (5% phenyl)-polymethylsiloxane stationary phase and a film thickness of 0.25 μm; He carrier gas with a column head pressure of 552 kPa and flow rate of 1.37 mL/min; injector temperature = 250 °C, ion source temperature = 200 °C. The GC oven temperature program was programmed to have an initial temperature of 50 °C, and the temperature increased at a rate of 2 °C/min to 260 °C. A 5% *w*/*v* solution of the sample in CH_2_Cl_2_ was prepared, and 0.1 μL was injected with a splitting mode (30:1). Identification of the oil components was based on their retention indices determined by reference to a homologous series of *n*-alkanes, and by comparison of their mass spectral fragmentation patterns with those reported in the databases [64,65,66,67]. The percentages of each component in the essential oils are reported as raw percentages based on total ion current without standardization. Gas chromatograms for the *Piper* essential oils are available as supplementary material.

### 3.3. Mosquito Larvicidal Assay

Eggs of *Ae. aegypti* were purchased from Institute of Biotechnology, Vietnam Academy of Science and Technology, and maintained at the Laboratory of Department of Pharmacy of Duy Tan University, Da Nang, Vietnam. For the assay, aliquots of the essential oils of *Piper* species, dissolved in DMSO (1% stock solution), was placed in a 500-mL beaker and added to water that contained 20 larvae (third and early fourth instar). With each experiment, a set of controls using DMSO was also run for comparison. Mortality was recorded after 24 h and again after 48 h of exposure during which no nutritional supplement was added. The experiments were carried out 25 ± 2 °C. Each test was conducted with four replicates with several concentrations (100, 50, 25, 12.5, 6.0, 3.0, 1.5, and 0.7 μg/mL). Larvicidal activities against *Ae. albopictus* and *Culex quinquefasciatus* (reared from wild populations) were determined similarly with concentrations of 150, 100, 50, 25, and 12.5 μg/mL. Permethrin was used as a positive control.

### 3.4. Statistical Analysis

Mosquito larvicidal activities (LC_50_ and LC_90_) were determined by log-probit analysis using XLSTAT v. 2018.5 (Addinsoft, Paris, France). The chemical compositions of the *Piper* essential oils were used in the multivariate analyses. The essential oil compositions were treated as operational taxonomic units (OTUs), and the concentrations (percentages) of 29 major essential oil components and the 24-h LC_50_ and LC_90_ larvicidal activity data were used to determine the associations between the *Piper* essential oils using agglomerative hierarchical cluster (AHC) analysis using XLSTAT Premium, version 2018.5 (Addinsoft, Paris, France). Dissimilarity was determined using Euclidean distance, and clustering was defined using Ward’s method. For the principal component analysis (PCA), the 29 major components and the larvicidal data were taken as variables using a Pearson correlation matrix using XLSTAT Premium, version 2018.5 (Addinsoft, Paris, France). A total of 414 data (31 variables × 12 samples) were used for the PCA.

## 4. Conclusions

Plant materials from 13 species of *Piper*, growing in central Vietnam were collected and the essential oils obtained and chemically analyzed. Larvicidal activity assays indicated that five *Piper* species (*P. nigrum*, *P. mutabile*, *P. longum*, *P. montium*, and *P. caninum*) showed excellent larvicidal activities with LC_50_ and LC_90_ values < 10 μg/mL, and that the larvicidal activities are correlated with concentrations of β-caryophyllene, β-bisabolene, β-pinene, and α-pinene. These Piper essential oils deserve consideration for future applications as natural, sustainable alternatives to synthetic pesticides for mosquito control.

## Figures and Tables

**Table 1:** Collection sites and essential oil yields for *Piper* essential oils from central Vietnam.

*Piper* Species	Voucher Numbers	Collection Site	Plant Parts	Essential Oil Yield (%) ^a^
*Piper arboricola* C. DC.	LTH 105	Ngoc Linh Nature Reserve-Quang Nam Province (15°50’16.0” N, 107°22’54.7” E, elev. 1341 m)	Leaves & stems	0.31
*Piper arboricola* C. DC.	LTH 105	Ngoc Linh Nature Reserve-Quang Nam Province (15°50’16.0” N, 107°22’54.7” E, elev. 1341 m)	Leaves	0.32
*Piper arboricola* C. DC.	LTH 105	Ngoc Linh Nature Reserve-Quang Nam Province (15°50’16.0” N, 107°22’54.7” E, elev. 1341 m)	Stems	0.12
*Piper bavinum* C. DC.	LTH 107	Ngoc Linh Nature Reserve-Quang Nam Province (15°50’16.0” N, 107°22’54.7” E, elev. 1341 m)	Leaves & stems	0.34
*Piper cambodianum* P. Fourn.	LTH 108	Ba Na-Nui Chua Nature Reserve (16°00′06.6″ N, 108°01′22.1″ E, elev. 791 m)	Leaves & stems	0.34
*Piper caninum* C. DC.	LTH 112	Chu Mom Ray National Park (14°25’33.5” N, 107°43’15.6” E, elev. 672 m)	Leaves & stems	0.10
*Piper longum* L.	LTH 109	Chu Mom Ray National Park (14°25’33.5” N, 107°43’15.6” E, elev. 672 m)	Leaves & stems	0.15
*Piper mekongense* C. DC	LTH 110	Chu Mom Ray National Park (14°25’33.5” N, 107°43’15.6” E, elev. 672 m)	Leaves & stems	0.32
*Piper montium* C. DC.	LTH 111	Chu Mom Ray National Park (14°25’33.5” N, 107°43’15.6” E, elev. 672 m)	Leaves & stems	0.10
*Piper mutabile* C. DC.	LTH 118	Chu Mom Ray National Park (14°25’33.5” N, 107°43’15.6” E, elev. 672 m)	Leaves & stems	0.38
*Piper nigrum* L.	LTH 113	Hoa Vang district, Da Nang city (16°01’0.6” N, 108°4’25.6” E, elev. 28 m)	Leaves & stems	0.39
*Piper politifolium* C. DC.	LTH 114	Chu Mom Ray National Park (14°25’33.5” N, 107°43’15.6” E, elev. 672 m)	Leaves & stems	0.37
*Piper rubrum* C. DC.	LTH 115	Ngoc Linh Nature Reserve-Quang Nam Province (15°50’16.0” N, 107°22’54.7” E, elev. 1341 m)	Leaves	0.41
*Piper rubrum* C. DC.	LTH 115	Ngoc Linh Nature Reserve-Quang Nam Province (15°50’16.0” N, 107°22’54.7” E, elev. 1341 m)	Stems	0.38
*Piper sarmentosum* Roxb.	LTH 116	Hoa Vang district, Da Nang city (16°01’0.6” N, 108°4’25.6” E, elev. 28 m)	Leaves & stems	0.29
*Piper umbellatum* L.	LTH 117	Chu Mom Ray National Park (14°25’33.5” N, 107°43’15.6” E, elev. 672 m)	Leaves & stems	0.025

^a^ Yields are based on mass of fresh plant material.

**Table 2:** Essential oil compositions (%) of *Piper* species from central Vietnam.

**RI ^a^**	**Compound**	***P. arboricola***	***P. arboricola***	***P. arboricola***	***P. bavinum***	***P. cambodianum***	***P. caninum***	***P. longum***	***P. mekongense***
		**Leaves & Stems**	**Leaves ^b^**	**Stems ^b^**	**Leaves & Stems**	**Leaves & Stems ^b^**	**Leaves & Stems ^b^**	**Leaves & Stems ^b^**	**Leaves & Stems ^b^**
900	Nonane	0.1	---	---	---	---	---	---	---
919	5,5-Dimethyl-1-vinylbicyclo[2.1.1]hexane	---	---	---	---	Tr ^c^	---	---	---
920	Tricyclene	---	---	---	---	---	---	---	tr
923	α-Thujene	0.1	tr	0.1	tr	tr	0.6	0.3	tr
930	α-Pinene	0.5	**6.4 ^d^**	**19.3**	3.3	4.1	**6.2**	**5.1**	3.8
945	α-Fenchene	---	---	---	---	---	---	---	tr
947	Camphene	0.6	0.6	0.7	0.3	0.1	1.1	1.0	1.4
970	Sabinene	0.7	tr	0.6	0.1	0.2	1.8	0.2	0.1
975	β-Pinene	0.1	0.8	**26.9**	3.9	4.6	**6.8**	**10.6**	**5.3**
982	6-Methylhept-5-en-2-one	---	---	---	---	---	---	---	---
986	Myrcene	**6.6**	0.5	1.1	0.2	2.7	0.7	0.8	0.2
997	δ-2-Carene	tr	---	---	---	---	---	---	tr
1002	*p*-Mentha-1(7),8-diene	---	---	---	---	tr	---	---	tr
1003	Octanal	---	---	---	---	---	---	---	---
1004	α-Phellandrene	tr	0.1	0.1	0.3	tr	0.1	0.1	tr
1007	δ-3-Carene	---	---	---	---	tr	---	0.1	tr
1010	Hexyl acetate	---	tr	---	---	---	---	---	---
1015	α-Terpinene	---	---	0.1	---	tr	0.4	0.2	---
1018	*m*-Cymene	---	---	---	---	---	---	---	---
1022	*p*-Cymene	0.2	1.3	0.5	tr	tr	0.6	0.6	0.2
1027	Limonene	**23.2**	2.4	**6.8**	2.2	**7.9**	1.6	1.4	0.8
1028	β-Phellandrene	0.1	0.2	0.4	0.2	0.2	3.4	0.1	0.1
1030	1,8-Cineole	0.1	tr	0.2	---	tr	0.4	0.2	0.1
1033	(*Z*)-β-Ocimene	0.3	0.1	---	0.3	tr	tr	0.1	tr
1035	2-Heptyl acetate	---	---	---	---	---	---	---	0.1
1043	(*E*)-β-Ocimene	0.1	tr	---	0.2	tr	0.2	1.5	0.1
1047	2,3,6-Trimethylhepta-1,5-diene	0.1	---	---	---	---	---	---	---
1055	γ-Terpinene	---	tr	0.2	0.1	0.2	1.5	1.4	---
1057	(2*E*)-Octenal	---	---	---	---	---	---	---	---
1067	*cis*-Sabinene hydrate	---	---	---	---	---	1.2	---	---
1079	*p*-Mentha-2,4(8)-diene	---	---	---	---	---	---	---	---
1083	Terpinolene	0.4	0.1	0.3	0.1	0.1	0.3	0.1	tr
1088	2-Nonanone	0.2	---	---	---	---	---	0.1	tr
1090	Rosefuran	---	---	---	---	---	---	---	---
1093	α-Pinene oxide	---	---	---	---	---	---	---	---
1097	Linalool	0.8	1.1	0.8	0.3	0.3	**10.1**	3.4	0.2
1098	*trans*-Sabinene hydrate	---	---	---	---	---	1.2	---	---
1098	2-Nonanol	tr	---	---	---	---	---	---	0.1
1101	6-Methyl-3,5-heptadien-2-one	---	---	---	---	---	---	---	---
1102	Nonanal	0.2	---	---	---	---	---	---	---
1103	1-Octen-3-yl acetate	tr	---	---	---	---	---	---	0.2
1105	*p*-Mentha-2-8-dien-1-ol	---	---	---	---	---	---	---	tr
1111	4,8-Dimethylnona-1,3,7-triene	0.3	tr	---	---	0.3	---	---	tr
1115	3-Octyl acetate	---	---	---	---	---	---	---	tr
1117	*trans-p*-Mentha-2,8-dien-1-ol	0.1	---	---	---	---	---	---	---
1122	*cis-p*-Menth-2-en-1-ol	---	---	---	---	---	0.4	---	---
1126	*allo*-Ocimene	---	---	---	---	---	---	---	---
1127	(*Z*)-Myroxide	---	---	---	---	---	---	---	---
1128	*cis*-Limonene oxide	tr	---	---	---	---	---	---	---
1129	Limona ketone	---	---	---	---	---	---	---	---
1132	*cis-p*-Mentha-2,8-dien-1-ol	0.1	---	---	---	---	---	---	---
1133	*trans*-Limonene oxide	tr	---	---	---	---	---	---	---
1135	Nopinone	---	---	---	---	---	---	---	tr
1137	(*E*)-Myroxide	0.1	---	---	---	---	---	---	---
1138	*trans*-Pinocarveol	---	---	---	---	---	---	---	0.1
1140	*trans-p*-Menth-2-en-1-ol	---	---	---	---	---	0.3	---	---
1143	*trans*-Verbenol	---	---	---	---	---	---	---	tr
1144	Camphor	tr	0.1	---	---	---	0.8	0.4	tr
1152	Camphene hydrate	---	---	---	---	---	---	tr	tr
1153	Sabina ketone	---	---	---	---	---	---	---	tr
1158	Isoborneol	0.1	---	---	---	---	---	---	---
1159	Pinocarvone	---	---	---	---	---	---	---	tr
1167	Rosefuran epoxide	---	---	---	---	---	---	---	---
1167	*p*-Mentha-1,5-dien-8-ol	---	---	---	---	---	---	---	---
1168	1-Nonanol	0.3	---	---	---	---	---	---	tr
1169	Borneol	---	tr	---	---	---	0.1	tr	tr
1171	(3*E*,5*E*)-Undeca-1,3,5-triene	---	---	---	---	---	---	---	---
1174	Isopinocamphone	---	---	---	---	---	---	0.1	---
1177	2-Isopropenyl-5-methyl-4-hexenal	---	---	---	---	---	---	tr	---
1178	Terpinen-4-ol	0.1	---	0.1	---	0.1	**7.7**	0.2	---
1182	Naphthalene	---	0.2	---	---	---	1.2	0.3	---
1184	*p*-Cymen-8-ol	0.4	---	---	---	---	---	---	0.1
1185	Cryptone	---	---	---	---	---	---	---	---
1193	Myrtenal	---	---	---	---	---	---	---	0.1
1193	α-Terpineol	---	tr	---	0.1	0.1	0.4	0.2	---
1199	(3*Z*)-Octenyl acetate	---	---	---	---	---	---	---	---
1201	*cis*-Sabinol	---	---	---	---	---	---	---	---
1203	Decanal	**6.2**	0.6	3.5	0.2	---	---	---	---
1206	(3*E*)-Octenyl acetate	---	---	---	---	---	---	---	---
1215	*trans*-Carveol	0.1	---	---	---	---	---	---	---
1216	*endo*-Fenchyl acetate	---	---	---	---	---	---	---	---
1221	2-Hydroxycineole	---	---	---	---	---	---	---	---
1222	Nerol	---	---	---	---	tr	---	---	---
1224	Citronellol	---	---	---	---	---	---	---	---
1225	Isobornyl formate	---	---	---	---	---	---	---	0.2
1228	*cis*-Carveol	tr	---	---	---	---	---	---	---
1228	2-Nonyl acetate	---	---	---	---	---	---	---	0.1
1233	Bornyl formate	---	---	---	---	---	---	---	1.6
1237	Ascaridole	---	---	---	---	---	0.7	0.1	---
1240	Carvone	0.1	---	---	---	---	---	---	---
1248	Geraniol	---	---	---	---	tr	---	---	---
1248	Linalyl acetate	---	0.1	---	---	---	---	---	---
1256	Methyl citronellate	---	---	---	---	---	---	---	---
1269	1-Decanol	1.1	tr	---	---	---	---	---	---
1271	Methyl hydrocinnamate	---	---	---	---	---	---	---	---
1277	9-Decen-1-ol	---	---	---	---	---	---	---	0.1
1281	Isobornyl acetate	---	---	0.2	---	---	---	0.1	0.1
1282	Bornyl acetate	---	1.3	---	0.1	---	0.7	0.3	0.2
1288	Safrole	---	0.1	---	0.5	---	---	---	---
1290	2-Undecanone	0.8	0.1	---	---	tr	0.4	1.7	0.2
1293	Methyl myrtenate	---	---	---	---	---	---	tr	---
1299	2-Undecanol	---	---	---	---	tr	---	tr	0.5
1300	Tridecane	---	0.1	0.4	---	---	1.2	0.1	---
1303	Undecanal	0.1	---	---	---	---	---	---	---
1304	Isoascaridole	---	---	---	---	---	0.5	0.1	---
1319	Methyl geranate	---	---	---	---	---	---	tr	---
1328	Myrtenyl acetate	---	tr	---	---	---	---	---	---
1328	Bicycloelemene	0.8	0.3	---	0.3	0.3	---	tr	0.3
1332	δ-Elemene	0.2	0.1	---	0.2	0.2	---	tr	0.7
1343	α-Cubebene	0.1	3.0	0.8	0.5	0.4	0.3	0.1	0.2
1344	α-Terpinyl acetate	---	---	---	---	---	---	---	---
1347	Citronellyl acetate	---	---	---	---	---	---	---	---
1358	Decanoic acid	0.1	---	---	---	---	---	---	---
1364	Cyclosativene	---	0.3	0.1	0.1	tr	---	0.1	0.2
1365	Hydrocinnamyl acetate	---	---	---	---	---	---	---	---
1366	α-Ylangene	---	---	---	---	---	---	---	---
1368	Isoledene	---	tr	---	0.2	tr	---	tr	tr
1372	α-Copaene	0.8	**13.7**	4.2	3.2	2.1	0.4	0.3	0.7
1375	Geranyl acetate	---	0.1	---	---	---	0.2	---	---
1376	(*E*)-β-Damascenone	---	---	---	---	---	---	tr	---
1380	β-Bourbonene	0.3	0.2	---	---	0.2	0.1	tr	0.1
1384	β-Cubebene	0.2	1.7	0.6	1.6	0.2	---	0.1	0.5
1385	7-*epi*-Sesquithujene	---	---	---	---	---	---	---	---
1386	β-Elemene	1.6	0.7	0.4	2.0	1.2	0.4	0.3	2.1
1398	Sesquithujene	---	tr	0.1	---	---	---	---	---
1402	Italicene	---	0.1	---	---	---	---	---	---
1402	(*Z*)-Caryophyllene	---	---	---	---	0.4	---	---	---
1403	α-Gurjunene	0.1	0.3	0.2	1.1	0.2	---	0.1	0.2
1406	Dodecanal	0.6	0.2	0.3	---	---	0.2	tr	---
1408	*cis*-5-Hydroxy-*p*-menth-6-en-2-one	---	---	---	---	---	---	---	---
1408	β-Maaliene	---	---	---	---	---	---	---	0.2
1409	*cis*-α-Bergamotene	---	0.3	1.0	0.4	---	0.2	tr	---
1416	β-Caryophyllene	**14.1**	**9.4**	4.9	**6.2**	**19.2**	**10.3**	**7.9**	4.0
1419	(*E*)-α-Ionone	---	tr	---	---	---	---	---	---
1422	γ-Maaliene	tr	---	---	---	tr	---	---	---
1423	γ-Elemene	---	---	---	---	---	---	---	0.9
1424	*trans*-5-Hydroxy-*p*-menth-6-en-2-one	---	---	---	---	---	---	---	---
1424	β-Gurjunene	---	---	---	---	---	---	---	2.1
1426	β-Copaene	0.3	0.7	0.8	0.4	0.2	0.4	0.1	---
1430	*trans*-α-Bergamotene	---	0.1	0.1	0.2	tr	0.1	tr	---
1430	α-Maaliene	0.1	---	---	---	---	---	tr	0.1
1431	α-Guaiene	---	0.7	0.1	0.5	0.3	---	---	---
1435	Aromadendrene	0.6	0.2	---	0.1	0.3	0.3	0.1	0.2
1438	(*Z*)-β-Farnesene	---	---	---	---	---	---	---	---
1439	6,9-Guaiadiene	---	---	---	---	---	---	---	---
1442	Myltayl-4(12)-ene	---	---	---	---	---	---	---	0.2
1443	Selina-5,11-diene	---	---	---	---	---	---	tr	---
1445	*cis*-Muurola-3,5-diene	---	0.9	0.3	---	0.1	---	tr	---
1445	Geranyl acetone	---	---	---	---	---	---	---	---
1446	*trans*-Muurola-3,5-diene	---	---	---	1.6	---	---	---	---
1449	(*E*)-β-Farnesene	---	0.2	0.3	0.5	tr	1.0	0.4	---
1450	Valerena-4,7(11)-diene	0.1	---	---	---	---	---	---	---
1452	α-Humulene	1.1	1.7	0.5	**6.1**	2.0	1.4	1.4	1.9
1453	Sesquisabinene	---	tr	0.2	0.1	---	---	---	---
1456	*allo*-Aromadendrene	0.1	0.1	---	0.2	0.4	0.1	---	---
1457	Eudesma-1,4(15),11-triene	---	---	---	---	---	---	---	---
1459	*cis*-Muurola-4(14),5-diene	---	---	---	---	---	---	0.1	---
1462	*cis*-Cadina-1(6),4-diene	---	---	---	---	tr	---	---	---
1464	Dehydroisolongifolenene	---	---	---	---	---	---	---	---
1467	Selina-4,11-diene	---	---	---	---	---	---	---	---
1467	(2*E*)-Undecenyl acetate	---	---	---	---	---	---	---	---
1468	*trans*-Cadina-1(6),4-diene	---	1.1	0.5	---	0.3	---	0.1	---
1470	γ-Gurjunene	---	---	---	1.7	---	---	---	---
1470	Amorpha-4,7(11)-diene	---	---	---	---	---	---	---	---
1471	γ-Muurolene	0.2	0.4	0.2	0.3	**9.6**	0.5	0.4	2.2
1472	β-Acoradiene	---	---	---	---	---	---	---	---
1473	γ-Curcumene	---	0.2	2.3	5.8	---	---	---	---
1475	*cis*-4,10-epoxy-Amorphane	---	---	---	---	0.6	---	---	---
1476	α-Amorphene	---	tr	---	---	---	---	---	---
1477	Germacrene D	2.8	---	---	4.7	1.5	---	1.2	5.2
1477	*ar*-Curcumene	---	5.5	9.6	---	---	4.9	---	---
1480	(*Z*,*Z*)-α-Farnesene	---	0.3	0.5	0.3	---	0.3	tr	---
1480	β-Chamigrene	---	---	---	---	0.9	---	---	1.3
1480	1-Dodecanol	---	---	---	---	---	---	---	---
1484	δ-Selinene	---	tr	---	---	---	---	---	---
1486	*trans*-Muurola-4(14),5-diene	---	1.6	0.6	1.4	---	---	0.3	---
1486	β-Selinene	0.1	0.2	1.1	1.6	0.6	1.4	tr	1.0
1487	Viridiflorene (=Ledene)	0.4	---	---	---	0.6	---	---	0.3
1488	Curzerene	---	---	---	---	---	---	---	0.2
1488	α-Zingiberene	---	---	---	---	---	0.8	---	---
1490	Unidentified sesquiterpenoid^e^	---	---	---	---	---	---	2.3	---
1492	Asaricin	---	---	---	---	---	---	---	---
1492	Bicyclogermacrene	**11.3**	**7.9**	---	**8.9**	3.7	---	1.5	**5.6**
1492	α-Selinene	---	---	---	---	---	1.1	---	---
1495	α-Muurolene	0.1	0.9	0.5	0.8	1.3	0.5	0.8	1.1
1495	*cis*-β-Guaiene	---	---	---	---	---	---	---	---
1497	(*Z*)-α-Bisabolene	---	---	---	---	---	---	0.1	---
1499	α-Bulnesene	---	2.5	0.7	3.0	1.2	---	---	---
1500	δ-Amorphene	---	---	---	---	---	---	---	---
1500	Pentadecane	---	---	0.5	---	---	---	---	---
1501	(*E*,*E*)-α-Farnesene	---	---	---	---	---	---	---	---
1504	β-Bisabolene	---	0.7	0.7	0.7	0.1	**8.7**	4.5	0.2
1505	β-Curcumene	---	0.1	0.3	1.5	---	---	---	---
1509	γ-Cadinene	0.1	0.2	---	0.2	0.5	0.5	1.3	0.6
1511	Cubebol	0.1	1.7	---	0.7	1.0	0.1	0.3	1.0
1514	δ-Cadinene	0.3	**6.0**	1.7	4.8	**6.1**	1.6	3.5	1.8
1515	7-*epi*-α-Selinene	---	---	---	---	---	---	---	1.4
1518	*trans*-Calamenene	0.1	**8.9**	2.4	1.1	0.2	---	---	0.2
1519	β-Sesquiphellandrene	---	---	0.2	0.2	---	1.2	---	---
1520	Zonarene	---	---	---	0.4	0.2	---	---	---
1523	(*E*)-γ-Bisabolene	---	---	---	0.2	---	---	---	---
1526	Kessane	---	---	---	---	0.2	---	---	---
1527	Nootkatene	---	0.1	---	---	---	---	---	---
1529	*trans*-Cadina-1,4-diene	---	0.8	0.3	0.9	0.7	---	0.1	---
1533	α-Cadinene	---	tr	---	---	0.1	---	0.3	0.2
1537	*cis*-Cadinene ether	---	tr	---	---	---	---	---	---
1538	(*E*)-α-Bisabolene	---	---	---	---	---	---	0.2	---
1538	α-Calacorene	---	0.4	---	---	0.1	---	---	0.2
1542	*trans*-Cadinene ether	---	0.5	---	---	---	---	0.2	---
1544	Unidentified sesquiterpenoid ^f^	0.3	---	---	---	---	---	1.3	---
1545	α-Elemol	---	---	---	---	0.1	---	---	0.3
1554	Germacrene B	0.1	---	---	---	---	---	---	2.0
1557	(*E*)-Nerolidol	1.7	0.4	---	0.2	1.6	2.3	**8.1**	0.9
1558	β-Calacorene	---	0.1	---	---	---	---	---	0.2
1565	Maaliol	---	---	---	---	---	---	---	**13.8**
1565	Palustrol	---	---	---	1.1	0.1	---	---	---
1566	Dendrolasin	---	---	---	---	---	---	---	---
1573	Spathulenol	**8.3**	0.6	---	0.4	1.3	1.2	0.7	**6.4**
1574	Germacra-1(10),5-dien-4β-ol	---	---	---	---	---	---	0.6	---
1578	Caryophyllene oxide	3.3	0.4	---	0.2	1.6	3.1	0.8	1.3
1582	Globulol	0.6	0.4	---	0.8	0.3	0.5	---	2.2
1583	Gleenol	---	0.3	---	---	---	---	---	---
1586	Unidentified sesquiterpenoid ^g^	---	0.1	---	---	0.1	---	---	---
1590	Viridiflorol	1.2	0.5	---	1.0	**6.5**	0.5	---	1.4
1593	Cubeban-11-ol	---	0.1	---	0.2	---	---	---	---
1595	Humulene epoxide I	---	---	---	---	---	---	---	---
1597	Curzerenone	---	---	---	---	---	---	---	---
1600	Rosifoliol	---	---	---	0.1	---	---	---	0.1
1600	Guaiol	---	---	---	---	0.8	---	---	---
1601	Ledol	---	0.1	---	0.9	0.3	---	---	---
1601	β-Oplopenone	---	---	---	---	---	---	0.5	---
1605	β-Atlantol	---	---	---	---	---	---	---	---
1606	Humulene epoxide II	0.2	0.1	---	---	0.1	0.3	---	0.3
1606	Tetradecanal	---	---	---	---	---	---	---	---
1609	Unidentified sesquiterpenoid ^h^	---	---	---	---	---	---	---	---
1612	1,10-di-*epi*-Cubenol	---	---	---	---	0.1	---	0.1	---
1613	α-Corocalene	---	0.1	---	---	---	---	---	---
1616	Junenol	---	---	---	---	---	---	---	---
1623	Selina-6-en-4β-ol	---	---	---	---	---	---	---	---
1624	1-*epi*-Cubenol	0.1	2.1	0.3	2.6	0.7	---	0.3	---
1626	*iso*-Spathulenol	0.7	---	---	---	0.3	---	---	1.3
1628	Caryophylla-4(12),8(13)-dien-5α-ol	---	---	---	---	---	---	---	---
1633	Caryophylla-4(12),8(13)-dien-5β-ol	---	---	---	---	0.2	---	---	---
1636	*allo*-Aromadendrene epoxide	---	---	---	---	---	---	---	---
1638	τ-Cadinol	---	---	---	---	0.9	0.4	2.2	1.1
1640	τ-Muurolol	0.2	---	---	0.6	0.6	0.5	2.0	2.9
1640	Cubenol	---	1.4	---	1.7	---	---	---	---
1643	α-Muurolol (= δ-Cadinol)	0.1	0.4	---	0.7	2.4	0.5	1.2	0.8
1648	Unidentified sesquiterpenoid ^i^	---	---	---	1.6	---	---	---	1.3
1651	α-Eudesmol	---	---	---	---	---	---	---	---
1652	α-Cadinol	---	0.2	---	1.2	1.8	0.8	4.9	3.6
1654	Selin-11-en-4α-ol	---	---	---	0.4	---	---	---	0.9
1655	Pogostol	---	0.4	---	0.4	0.2	---	---	---
1656	Unidentified fatty acid derivative ^j^	0.2	---	---	---	---	---	---	---
1658	*ar*-Turmerone	---	0.2	---	---	---	---	---	---
1658	9-Methoxycalamenene	---	---	---	---	---	---	---	0.3
1661	Intermedeol	---	---	---	---	---	---	---	---
1661	Bulnesol	---	---	---	---	0.3	---	---	---
1663	(6*Z*)-Pentadecen-2-one	---	---	---	---	---	---	---	---
1664	(2*E*,6*Z*)-Farnesol	---	---	---	---	---	---	---	---
1666	14-Hydroxy-9-*epi*-(*E*)-caryophyllene	---	---	---	---	0.1	---	---	---
1670	Cadalene	---	0.1	---	---	---	---	---	---
1680	Unidentified sesquiterpenoid ^k^	---	---	---	---	---	---	**5.7**	---
1680	Germacra-4(15),5,10(14)-trien-1α-ol	---	---	---	---	tr	---	---	---
1681	*epi*-α-Bisabolol		---	---		---	---	---	2.4
1684	α-Bisabolol	---	---	---	---	---	---	---	---
1686	(2*Z*,6*Z*)-Farnesol	---	---	---	---	---	---	---	---
1690	Shyobunol	---	---	---	---	1.5	---	---	---
1690	2-Pentadecanone	---	---	---	---	---	---	---	---
1700	Heptadecane	---	---	---	---	---	---	---	---
1710	Unidentified sesquiterpenoid^l^	---	---	---	---	---	---	**11.9**	---
1711	(2*E*,6*Z*)-Farnesol	---	---	---	---	---	---	---	---
1722	Cryptomerione	---	---	---	---	---	---	---	---
1725	Zerumbone	---	---	---	---	---	---	---	---
1747	Cyclocolorenone	0.5	0.1	0.1	**7.9**	---	---	---	---
1763	Benzyl benzoate	---	---	---	---	0.1	---	---	---
1798	Curzerenone B	---	---	---	---	---	---	---	---
1809	Hexadecanal	---	---	---	---	---	---	---	---
1828	(2*Z*,6*E*)-Farnesyl acetate	---	---	---	---	---	---	---	---
2017	(*E*,*E*)-Geranyl linalool	---	---	---	---	---	---	---	---
2103	(*E*)-Phytol	---	---	---	---	---	---	---	---
	Monoterpene hydrocarbons	33.0	12.5	57.2	11.0	20.0	25.3	23.7	11.9
	Oxygenated monoterpenoids	1.8	2.7	1.3	0.5	0.4	24.6	5.1	2.6
	Sesquiterpene hydrocarbons	35.9	73.0	36.3	64.1	55.2	36.5	25.3	38.4
	Oxygenated sesquiterpenoids	17.0	9.7	0.4	20.9	23.4	10.1	21.9	41.0
	Diterpenoids	0.0	0.0	0.0	0.0	0.0	0.0	0.0	0.0
	Benzenoids	0.0	0.1	0.0	0.5	0.1	0.0	0.0	0.0
	Others	9.9	1.1	4.8	0.2	0.3	3.0	2.1	1.3
	Total identified	97.6	99.0	100.0	97.1	99.5	99.4	78.2	95.2
**RI ^a^**	**Compound**	***P. montium***	***P. mutabile***	***P. nigrum***	***P. politifolium***	***P. rubrum***	***P. rubrum***	***P. sarmentosum***	***P. umbellatum***
		**Leaves & Stems ^b^**	**Leaves & Stems ^b^**	**Leaves & Stems ^b^**	**Leaves & Stems**	**Leaves ^b^**	**Stems ^b^**	**Leaves & Stems ^b^**	**Leaves & Stems**
900	Nonane	---	---	---	---	---	---	---	---
919	5,5-Dimethyl-1-vinylbicyclo[2.1.1]hexane	---	---	---	---	---	---	---	---
920	Tricyclene	---	---	---	tr ^c^	---	---	tr	---
923	α-Thujene	tr	---	tr	0.8	tr	0.1	0.1	---
930	α-Pinene	0.2	3.1	0.8	**12.9** ^d^	0.9	0.5	2.3	0.4
945	α-Fenchene	---	---	tr	---	---	---	tr	---
947	Camphene	tr	1.0	tr	3.2	0.2	0.8	2.0	0.1
970	Sabinene	---	---	tr	1.7	0.2	2.1	0.1	0.1
975	β-Pinene	0.2	**5.5**	1.3	**7.1**	0.1	0.3	**5.5**	0.8
982	6-Methylhept-5-en-2-one	---	---	---	---	---	---	tr	---
986	Myrcene	tr	0.2	0.5	1.6	4.1	0.9	0.4	0.3
997	δ-2-Carene	---	---	---	---	tr	---	---	---
1002	*p*-Mentha-1(7),8-diene	---	---	---	---	---	---	---	tr
1003	Octanal	0.1	---	---	---	---	---	---	---
1004	α-Phellandrene	0.2	---	1.0	0.4	---	0.1	---	tr
1007	δ-3-Carene	---	0.2	4.6	2.3	---	---	---	tr
1010	Hexyl acetate	---	---	---	---	---	---	---	---
1015	α-Terpinene	tr	---	tr	0.4	---	0.1	---	---
1018	*m*-Cymene	---	---	tr	---	---	---	---	---
1022	*p*-Cymene	0.2	---	0.1	0.3	0.2	0.2	0.1	tr
1027	Limonene	0.4	3.8	2.4	2.6	**7.0**	**9.3**	1.3	0.9
1028	β-Phellandrene	0.2	0.4	0.1	1.4	tr	0.8	tr	0.5
1030	1,8-Cineole	0.2	0.2	---	---	---	---	0.5	---
1033	(*Z*)-β-Ocimene	tr	---	tr	0.4	0.2	tr	0.3	4.3
1035	2-Heptyl acetate	---	---	---	---	---	---	0.1	---
1043	(*E*)-β-Ocimene	tr	0.2	0.2	2.8	tr	0.1	1.3	1.0
1047	2,3,6-Trimethylhepta-1,5-diene	---	---	---	---	---	---	---	---
1055	γ-Terpinene	0.1	---	0.1	0.6	---	0.1	---	tr
1057	(2*E*)-Octenal	0.2	---	---	---	---	---	---	---
1067	*cis*-Sabinene hydrate	tr	---	---	---	---	---	---	---
1079	*p*-Mentha-2,4(8)-diene	---	---	0.1	---	---	---	---	---
1083	Terpinolene	tr	0.2	0.2	**5.7**	0.1	0.6	---	tr
1088	2-Nonanone	---	---	---	---	---	0.2	0.1	---
1090	Rosefuran	---	---	---	---	---	---	0.1	---
1093	α-Pinene oxide	---	---	---	---	---	---	0.1	---
1097	Linalool	2.1	3.7	0.8	0.8	0.4	0.3	0.4	0.5
1098	*trans*-Sabinene hydrate	---	---	---	---	---	---	---	---
1098	2-Nonanol	---	---	---	---	---	0.1	---	---
1101	6-Methyl-3,5-heptadien-2-one	---	---	---	---	---	---	0.2	---
1102	Nonanal	---	---	---	---	0.1	0.3	---	---
1103	1-Octen-3-yl acetate	---	---	---	---	---	---	---	0.4
1105	*p*-Mentha-2-8-dien-1-ol	---	---	---	---	---	---	---	---
1111	4,8-Dimethylnona-1,3,7-triene	---	---	---	---	0.7	0.5	tr	tr
1115	3-Octyl acetate	---	---	---	---	---	---	---	0.1
1117	*trans-p*-Mentha-2,8-dien-1-ol	---	---	---	---	---	---	---	tr
1122	*cis-p*-Menth-2-en-1-ol	---	---	---	---	---	---	---	---
1126	*allo*-Ocimene	---	---	---	---	---	---	tr	0.2
1127	(*Z*)-Myroxide	---	---	---	---	---	---	tr	---
1128	*cis*-Limonene oxide	---	---	---	---	---	---	---	---
1129	Limona ketone	---	---	---	---	---	---	tr	---
1132	*cis-p*-Mentha-2,8-dien-1-ol	---	---	---	---	---	---	---	---
1133	*trans*-Limonene oxide	---	---	---	---	---	---	---	---
1135	Nopinone	---	---	---	---	---	---	---	---
1137	(*E*)-Myroxide	---	---	---	---	---	---	0.1	---
1138	*trans*-Pinocarveol	---	---	---	---	---	---	0.1	---
1140	*trans-p*-Menth-2-en-1-ol	---	---	---	---	---	---	---	---
1143	*trans*-Verbenol	---	---	---	---	---	---	tr	---
1144	Camphor	tr	1.0	---	---	---	---	tr	---
1152	Camphene hydrate	---	---	---	---	---	---	---	---
1153	Sabina ketone	---	---	---	---	---	---	---	---
1158	Isoborneol	---	---	---	---	---	---	---	---
1159	Pinocarvone	---	---	---	---	---	---	tr	---
1167	Rosefuran epoxide	---	---	---	---	---	---	tr	---
1167	*p*-Mentha-1,5-dien-8-ol	---	---	---	---	---	---	---	tr
1168	1-Nonanol	---	---	---	---	0.1	0.2	---	---
1169	Borneol	---	0.1	---	---	---	---	0.1	---
1171	(3*E*,5*E*)-Undeca-1,3,5-triene	---	---	---	---	---	---	---	tr
1174	Isopinocamphone	---	---	---	---	---	---	---	---
1177	2-Isopropenyl-5-methyl-4-hexenal	---	0.2	---	---	---	---	---	---
1178	Terpinen-4-ol	0.8	0.3	---	0.4	---	0.1	tr	---
1182	Naphthalene	0.8	2.3	---	---	---	0.3	---	0.2
1184	*p*-Cymen-8-ol	---	---	---	0.1	0.2	0.2	tr	---
1185	Cryptone	---	---	---	---	---	---	tr	---
1193	Myrtenal	---	---	---	---	---	---	0.2	---
1193	α-Terpineol	0.1	0.3	tr	0.1	---	---	---	---
1199	(3*Z*)-Octenyl acetate	---	---	---	---	---	---	tr	0.1
1201	*cis*-Sabinol	---	---	---	---	---	---	---	---
1203	Decanal	---	---	---	---	1.3	**31.6**	---	---
1206	(3*E*)-Octenyl acetate	---	---	---	---	---	---	0.1	0.1
1215	*trans*-Carveol	---	---	---	---	---	---	---	---
1216	*endo*-Fenchyl acetate	---	---	---	---	---	---	0.1	---
1221	2-Hydroxycineole	---	---	---	---	---	---	---	---
1222	Nerol	---	---	---	---	---	---	---	---
1224	Citronellol	---	---	tr	---	---	---	---	---
1225	Isobornyl formate	---	---	---	---	---	---	---	---
1228	*cis*-Carveol	---	---	---	---	---	---	---	---
1228	2-Nonyl acetate	---	---	---	---	---	---	---	---
1233	Bornyl formate	---	---	---	---	---	---	---	tr
1237	Ascaridole	---	---	---	---	---	---	---	---
1240	Carvone	---	---	---	---	---	---	---	---
1248	Geraniol	---	---	---	---	---	---	---	---
1248	Linalyl acetate	---	---	0.1	---	---	---	---	---
1256	Methyl citronellate	---	---	0.5	---	---	---	---	---
1269	1-Decanol	0.2	---	---	---	0.1	3.4	---	---
1271	Methyl hydrocinnamate	---	---	---	---	---	---	0.1	---
1277	9-Decen-1-ol	---	---	---	---	---	---	---	---
1281	Isobornyl acetate	0.1	---	---	---	---	---	1.6	0.2
1282	Bornyl acetate	---	---	---	0.1	0.1	---	0.1	tr
1288	Safrole	---	---	---	2.7	---	---	---	0.1
1290	2-Undecanone	0.1	---	0.2	1.6	0.2	2.3	0.3	tr
1293	Methyl myrtenate	---	---	---	---	---	---	---	---
1299	2-Undecanol	---	---	tr	---	---	0.1	---	tr
1300	Tridecane	2.8	1.9	---	---	---	0.5	---	---
1303	Undecanal	---	---	---	---	---	0.1	---	---
1304	Isoascaridole	---	---	---	---	---	---	---	---
1319	Methyl geranate	---	---	0.1	---	---	---	---	---
1328	Myrtenyl acetate	0.1	---	---	---	---	---	---	---
1328	Bicycloelemene	---	---	1.6	0.4	1.1	0.3	---	0.9
1332	δ-Elemene	0.1	0.2	**20.4**	0.2	0.1	0.1	0.4	**9.2**
1343	α-Cubebene	0.2	0.1	1.1	0.1	0.4	0.1	---	0.1
1344	α-Terpinyl acetate	---	---	---	---	---	---	0.3	---
1347	Citronellyl acetate	---	---	0.1	---	---	---	---	---
1358	Decanoic acid	---	---	---	---	---	0.4	---	---
1364	Cyclosativene	0.2	0.3	0.1	---	0.1	---	---	tr
1365	Hydrocinnamyl acetate	---	---	---	---	---	---	0.5	---
1366	α-Ylangene	0.1	---	0.1	---	---	---	---	---
1368	Isoledene	---	---	0.3	---	0.1	---	---	---
1372	α-Copaene	0.7	1.3	3.5	1.1	**7.1**	1.7	0.2	0.4
1375	Geranyl acetate	0.2	---	---	---	---	---	---	---
1376	(*E*)-β-Damascenone	---	---	---	---	---	---	---	---
1380	β-Bourbonene	0.1	---	0.1	0.1	0.4	0.1	0.1	tr
1384	β-Cubebene	---	---	---	0.2	0.4	0.2	---	0.1
1385	7-*epi*-Sesquithujene	---	---	---	---	---	---	0.1	---
1386	β-Elemene	1.2	0.4	3.3	0.6	2.8	0.5	2.0	0.6
1398	Sesquithujene	---	---	---	---	---	---	---	---
1402	Italicene	---	---	---	---	---	---	---	---
1402	(*Z*)-Caryophyllene	---	---	tr	---	---	---	---	tr
1403	α-Gurjunene	tr	0.2	2.2	0.1	0.2	0.1	---	0.1
1406	Dodecanal	0.1	---	---	---	0.3	**5.0**	---	---
1408	*cis*-5-Hydroxy-*p*-menth-6-en-2-one	---	---	---	---	---	---	---	---
1408	β-Maaliene	---	---	0.4	---	---	---	---	---
1409	*cis*-α-Bergamotene	0.4	0.2	---	---	0.1	---	0.1	---
1416	β-Caryophyllene	**19.2**	**16.5**	**7.7**	**6.5**	**22.9**	**9.7**	**5.5**	**44.8**
1419	(*E*)-α-Ionone	---	---	---	---	---	---	---	---
1422	γ-Maaliene	---	---	tr	---	0.1	---	---	tr
1423	γ-Elemene	---	---	0.2	---	---	---	---	0.2
1424	*trans*-5-Hydroxy-*p*-menth-6-en-2-one	---	---	---	---	---	---	---	---
1424	β-Gurjunene	---	---	---	---	---	---	---	---
1426	β-Copaene	0.6	0.9	---	0.2	0.5	0.2	0.1	0.3
1430	*trans*-α-Bergamotene	0.2	0.2	---	0.1	---	---	0.1	tr
1430	α-Maaliene	---	---	---	---	0.1	---	---	tr
1431	α-Guaiene	---	---	---	0.2	---	---	---	---
1435	Aromadendrene	0.2	0.4	0.9	0.1	1.3	0.2	0.1	0.1
1438	(*Z*)-β-Farnesene	---	---	---	---	---	---	0.1	---
1439	6,9-Guaiadiene	---	---	0.5	---	---	---	---	0.2
1442	Myltayl-4(12)-ene	---	0.1	---	---	0.1	---	---	tr
1443	Selina-5,11-diene	---	---	---	---	---	---	---	---
1445	*cis*-Muurola-3,5-diene	0.1	0.1	0.1	---	---	---	---	0.2
1445	Geranyl acetone	---	---	0.4	---	---	---	---	0.4
1446	*trans*-Muurola-3,5-diene	---	---	---	---	---	---	---	---
1449	(*E*)-β-Farnesene	1.6	1.1	---	---	---	---	2.2	0.1
1450	Valerena-4,7(11)-diene	---	---	tr	---	0.1	---	---	---
1452	α-Humulene	1.6	2.1	2.7	3.2	1.9	0.8	1.2	3.8
1453	Sesquisabinene	---	---	---	---	---	---	---	---
1456	*allo*-Aromadendrene	0.1	---	---	---	0.2	---	---	0.1
1457	Eudesma-1,4(15),11-triene	---	---	0.6	---	---	---	---	---
1459	*cis*-Muurola-4(14),5-diene	---	---	---	---	---	---	---	0.1
1462	*cis*-Cadina-1(6),4-diene	---	---	0.4	---	---	---	---	---
1464	Dehydroisolongifolenene	---	---	---	---	---	---	---	0.1
1467	Selina-4,11-diene	---	---	---	---	---	---	---	0.1
1467	(2*E*)-Undecenyl acetate	---	---	---	---	---	---	0.1	---
1468	*trans*-Cadina-1(6),4-diene	0.2	---	---	---	---	---	---	---
1470	γ-Gurjunene	---	---	0.6	---	0.1	---	---	---
1470	Amorpha-4,7(11)-diene	---	---	---	---	---	---	---	1.7
1471	γ-Muurolene	1.0	1.0	3.6	0.1	0.2	0.3	---	0.4
1472	β-Acoradiene	---	---	---	---	---	---	0.7	---
1473	γ-Curcumene	---	---	---	---	---	---	---	---
1475	*cis*-4,10-epoxy-Amorphane	---	---	---	---	---	---	---	---
1476	α-Amorphene	---	0.2	0.2	---	---	---	---	---
1477	Germacrene D	---	---	1.1	5.1	2.9	1.8	---	1.8
1477	*ar*-Curcumene	**15.1**	**8.2**	---	---	---	---	1.8	---
1480	(*Z*,*Z*)-α-Farnesene	0.8	0.7	---	---	---	---	0.5	---
1480	β-Chamigrene	---	---	---	---	0.1	---	---	---
1480	1-Dodecanol	---	---	---	---	---	0.5	---	---
1484	δ-Selinene	---	---	2.2	---	---	---	---	1.3
1486	*trans*-Muurola-4(14),5-diene	0.4	0.3	2.0	---	---	---	---	0.2
1486	β-Selinene	1.4	0.7	**5.1**	0.6	0.3	0.1	4.4	0.3
1487	Viridiflorene (=Ledene)	---	---	2.5	0.1	0.9	0.3	---	---
1488	Curzerene	---	---	---	---	---	---	---	---
1488	α-Zingiberene	3.1	2.2	---	---	---	---	---	---
1490	Unidentified sesquiterpenoid ^e^	---	---	---	---	---	---	---	---
1492	Asaricin	---	3.2	---	**18.7**	---	---	---	---
1492	Bicyclogermacrene	---	---	---	---	**15.7**	3.5	---	1.6
1492	α-Selinene	0.9	---	4.3	---	---	---	3.2	---
1495	α-Muurolene	1.5	1.1	0.2	0.3	0.3	0.1	0.1	0.3
1495	*cis*-β-Guaiene	---	---	---	---	---	---	---	0.2
1497	(*Z*)-α-Bisabolene	---	---	---	---	---	---	0.1	---
1499	α-Bulnesene	---	---	---	---	0.1	---	---	---
1500	δ-Amorphene	---	---	0.2	---	---	---	---	---
1500	Pentadecane	---	---	---	---	0.2	**5.3**	---	---
1501	(*E*,*E*)-α-Farnesene	---	---	0.4	0.1	---	---	---	---
1504	β-Bisabolene	**22.1**	**12.9**	0.3	0.1	0.1	---	**40.3**	0.6
1505	β-Curcumene	---	---	---	---	---	---	---	---
1509	γ-Cadinene	0.6	0.7	0.2	0.1	0.1	---	---	0.4
1511	Cubebol	0.1	---	0.2	0.1	0.1	0.1	---	0.2
1514	δ-Cadinene	2.1	2.8	1.4	1.5	0.9	0.5	---	1.2
1515	7-*epi*-α-Selinene	---	---	---	---	---	---	0.2	---
1518	*trans*-Calamenene	0.3	0.4	0.2	tr	1.1	0.2	---	0.5
1519	β-Sesquiphellandrene	2.9	2.1	---	0.1	---	---	0.2	0.1
1520	Zonarene	---	---	---	---	---	---	---	---
1523	(*E*)-γ-Bisabolene	0.2	0.1	---	---	---	---	---	---
1526	Kessane	---	---	---	---	---	---	---	---
1527	Nootkatene	---	---	---	---	---	---	---	---
1529	*trans*-Cadina-1,4-diene	0.2	0.2	0.7	---	---	---	---	0.1
1533	α-Cadinene	0.2	0.2	0.1	---	---	---	---	0.2
1537	*cis*-Cadinene ether	---	---	---	---	---	---	---	---
1538	(*E*)-α-Bisabolene	---	---	---	---	---	---	0.5	---
1538	α-Calacorene	0.3	0.3	0.1	---	---	---	---	0.2
1542	*trans*-Cadinene ether	---	---	---	---	---	---	---	---
1544	Unidentified sesquiterpenoid ^f^	---	---	---	---	---	---	---	---
1545	α-Elemol	0.1	---	0.1	---	---	---	0.2	---
1554	Germacrene B	---	---	0.2	0.1	---	---	---	0.2
1557	(*E*)-Nerolidol	---	---	0.2	0.7	2.6	4.7	0.7	4.1
1558	β-Calacorene	---	---	---	---	---	---	---	---
1565	Maaliol	---	0.3	---	0.2	---	---	---	1.0
1565	Palustrol	---	---	0.1	0.1	---	---	---	---
1566	Dendrolasin	---	---	---	---	---	---	tr	---
1573	Spathulenol	0.5	1.0	0.3	1.0	**8.6**	1.1	1.3	---
1574	Germacra-1(10),5-dien-4β-ol	---	---	---	---	---	---	---	0.9
1578	Caryophyllene oxide	3.1	3.5	0.2	0.5	3.8	1.3	**5.1**	2.5
1582	Globulol	0.2	0.4	0.3	0.2	0.5	---	0.1	0.3
1583	Gleenol	---	---	---	---	---	---	---	---
1586	Unidentified sesquiterpenoid ^g^	---	---	3.2	---	---	---	---	0.1
1590	Viridiflorol	0.2	0.2	0.1	0.2	1.0	0.2	tr	0.2
1593	Cubeban-11-ol	---	---	---	---	---	---	---	---
1595	Humulene epoxide I	---	---	---	---	---	---	tr	---
1597	Curzerenone	0.4	---	---	---	---	---	---	---
1600	Rosifoliol	---	---	---	tr	---	---	---	tr
1600	Guaiol	---	---	0.1	---	---	---	---	tr
1601	Ledol	---	---	---	0.1	---	---	---	---
1601	β-Oplopenone	---	---	---	---	---	---	---	---
1605	β-Atlantol	---	---	0.5	---	---	---	---	---
1606	Humulene epoxide II	0.2	0.2	---	0.1	0.2	0.1	0.8	0.1
1606	Tetradecanal	---	---	---	---	---	0.4	---	---
1609	Unidentified sesquiterpenoid ^h^	---	2.8	---	---	---	---	---	---
1612	1,10-di-*epi*-Cubenol	---	---	---	---	---	---	---	---
1613	α-Corocalene	---	---	---	---	---	---	---	---
1616	Junenol	---	---	---	---	---	---	---	---
1623	Selina-6-en-4β-ol	---	---	0.7	---	---	---	---	0.9
1624	1-*epi*-Cubenol	0.5	0.4	---	0.1	0.2	0.3	---	---
1626	*iso*-Spathulenol	---	---	3.9	---	0.5	0.1	0.5	2.5
1628	Caryophylla-4(12),8(13)-dien-5α-ol	---	---	---	---	---	---	0.4	---
1633	Caryophylla-4(12),8(13)-dien-5β-ol	---	---	---	---	---	---	0.2	0.2
1636	*allo*-Aromadendrene epoxide	---	---	---	---	---	---	---	---
1638	τ-Cadinol	0.4	0.3	---	0.1	---	0.2	---	0.4
1640	τ-Muurolol	0.8	1.2	---	0.1	---	0.1	---	0.5
1640	Cubenol	---	---	---	---	0.3	---	---	---
1643	α-Muurolol (= δ-Cadinol)	0.9	1.3	---	0.2	0.2	0.3	0.2	0.1
1648	Unidentified sesquiterpenoid ^i^	---	---	---	0.1	---	---	---	---
1651	α-Eudesmol	---	---	---	---	---	---	0.2	---
1652	α-Cadinol	1.6	1.2	0.3	0.3	---	0.3	---	0.7
1654	Selin-11-en-4α-ol	---	---	0.1	---	---	0.3	---	---
1655	Pogostol	0.5	---	---	---	---	---	0.3	---
1656	Unidentified fatty acid derivative^j^	---	1.0	---	3.9	---	---	---	---
1658	*ar*-Turmerone	---	---	---	---	---	0.1	---	---
1658	9-Methoxycalamenene	---	---	---	---	---	---	---	---
1661	Intermedeol	---	---	tr	---	---	---	0.2	---
1661	Bulnesol	---	---	---	---	---	---	---	---
1663	(6*Z*)-Pentadecen-2-one	---	---	---	0.2	---	0.4	---	---
1664	(2*E*,6*Z*)-Farnesol	---	---	---	---	---	---	---	0.2
1666	14-Hydroxy-9-*epi*-(*E*)-caryophyllene	---	---	---	---	---	---	---	---
1670	Cadalene	---	---	---	---	---	---	---	---
1680	Unidentified sesquiterpenoid ^k^	---	---	---	---	0.2	---	---	---
1680	Germacra-4(15),5,10(14)-trien-1α-ol	---	---	---	---	---	---	---	---
1681	*epi*-α-Bisabolol	---	---	---	---	---	---	0.1	---
1684	α-Bisabolol	---	---	---	---	---	---	0.2	0.2
1686	(2*Z*,6*Z*)-Farnesol	---	---	---	---	---	---	0.3	---
1690	Shyobunol	---	---	---	---	---	---	---	---
1690	2-Pentadecanone	---	---	---	0.1	---	0.3	---	---
1700	Heptadecane	---	---	---	---	---	0.3	---	---
1710	Unidentified sesquiterpenoid ^l^	---	---	---	---	---	---	---	---
1711	(2*E*,6*Z*)-Farnesol	---	---	0.2	---	---	---	0.2	---
1722	Cryptomerione	---	---	---	---	---	---	0.3	---
1725	Zerumbone	---	---	---	---	---	---	0.1	---
1747	Cyclocolorenone	---	---	---	0.5	tr	0.1	---	---
1763	Benzyl benzoate	---	---	---	---	---	---	---	---
1798	Curzerenone B	0.3	---	---	---	---	---	---	---
1809	Hexadecanal	---	---	---	---	---	0.2	---	---
1828	(2*Z*,6*E*)-Farnesyl acetate	---	---	0.1	---	---	---	0.1	---
2017	(*E*,*E*)-Geranyl linalool	---	---	---	---	---	---	0.1	---
2103	(*E*)-Phytol	---	---	0.1	---	---	---	0.3	0.9
	Monoterpene hydrocarbons	1.5	14.6	11.4	44.3	13.0	15.9	13.2	8.5
	Oxygenated monoterenoids	3.5	5.9	1.5	1.4	0.6	0.6	3.6	0.7
	Sesquiterpene hydrocarbons	79.9	58.2	71.9	21.4	62.9	20.9	64.1	72.6
	Oxygenated sesquiterpenoids	10.0	10.1	7.4	4.5	18.1	9.3	11.3	15.0
	Diterpenoids	0.0	0.0	0.1	0.0	0.0	0.0	0.4	0.9
	Benzenoids	0.0	3.2	0.0	21.4	0.0	0.0	0.6	0.1
	Others	4.3	4.2	0.5	1.9	2.9	52.4	0.9	1.3
	Total identified	99.2	96.2	92.8	94.8	97.5	99.0	94.2	99.0

^a^ RI = “Retention Index” determined in reference to a homologous series of *n*-alkanes. ^b^ Essential oil was used for larvicidal activity screening. ^c^ tr = “trace” (<0.05%). ^d^ Major essential oil components (>5%) are highlighted in **blue bold** font. ^e^ MS(EI): 202(45%), 187(18%), 159(70%), 145(47%), 131(58%), 119(60%), 117(60%), 105(71%), 91(100%), 81(55%), 43(100%). ^f^ MS(EI): 218(8%), 176(13%), 161(17%), 134(32%), 119(100%), 105(48%), 91(38%), 77(18%), 55(16%), 41(27%). ^g^ MS(EI): 220(11%), 205(10%), 187(9%), 162(63%), 147(65%), 134(41%), 119(100%), 107(42%), 105(53%), 93(67%), 91(58%), 79(29%), 69(18%), 55(21%), 43(100%), 41(28%). ^h^ MS(EI): 204(56%), 161(30%), 148(18%), 135(25%), 131(100%), 122(14%), 117(18%), 115(19%), 103(34%), 91(11%), 77(23%), 51(11%), 41(7%). ^i^ MS(EI): 223(8%), 162(97%), 147(67%), 133(65%), 119(60%), 105(100%), 94(81%), 91(68%), 81(52%), 67(25%), 59(88%), 43(34%), 41(33%). ^j^ MS(EI): 164(13%), 95(20%), 94(21%), 93(28%), 91(21%), 81(38%), 80(65%), 79(100%), 67(56%), 55(27%), 54(18%), 43(98%), 41(38%). ^k^ MS(EI): 218(51%), 190(14%), 175(30%), 161(79%), 147(49%), 134(87%), 119(100%), 105(53%), 91(49%), 77(21%), 55(17%), 41(34%). ^l^ MS(EI): 218(6%), 203(8%), 176(40%), 161(100%), 147(28%), 124(65%), 121(26%), 119(27%), 105(31%), 95(44%), 91(30%), 77(20%), 67(31%), 55(17%), 41(37%).

**Table 3:** Major essential oil components (%) of *Piper umbellatum*.

	Vietnam	Belém-1	Belém-2	Belém-3	Belém-4	Campinas	Monteverde	Cuba	Araraquara
Compound	This work	[50]	[50]	[50]	[50]	[51]	[53]	[54]	[52]
Camphor	0.0	0.0	0.3	4.2	0.0	0.0	0.0	9.6	0.0
Safrole	0.1	0.0	0.0	0.0	0.0	0.0	0.0	26.4	0.0
δ-Elemene	9.2	0.0	0.0	0.0	0.0	1.5	1.4	0.0	0.0
β-Elemene	0.6	0.0	0.0	0.0	0.0	0.0	6.9	0.0	0.0
β-Caryophyllene	44.8	68.0	39.1	39.5	64.8	6.3	28.3	6.6	3.0
α-Humulene	3.8	5.9	4.0	2.1	6.5	0.7	2.2	0.0	1.1
γ-Muurolene	0.4	0.0	0.0	0.0	0.0	0.5	0.0	0.5	8.9
Germacrene D	1.8	6.2	13.3	12.7	8.7	55.8	16.7	3.7	34.2
Bicyclogermacrene	1.6	2.2	5.8	4.3	3.2	11.8	6.6	0.5	9.0
γ-Cadinene	0.4	0.4	1.4	2.1	0.0	1.4	0.0	0.0	5.9
δ-Cadinene	1.2	1.0	3.8	2.2	1.0	2.0	0.2	2.0	15.0
(*E*)-Nerolidol	4.1	4.9	4.8	11.1	7.5	0.4	4.4	4.9	4.4

**Table 4:** Larvicidal activities (LC_50_ and LC_90_, μg/mL) of *Piper* essential oils from central Vietnam.

**Mosquito Species**	***Piper* species**						
	***P. arboricola***	***P. arboricola***	***P. cambodianum***	***P. caninum***	***P. longum***	***P. mekongense***	***P. montium***
	**Leaves**	**Stems**	**Leaves & Stems**	**Leaves & Stems**	**Leaves & Stems**	**Leaves & Stems**	**Leaves & Stems**
*Aedes aegypti*							
24-h LC_50_ (95% confidence)	26.85 (24.55–29.52)	28.45 (26.54–30.77)	8.861 (7.012–10.650)	1.376 (1.252–1.513)	5.342 (4.950–5.757)	40.16 (37.09–42.88)	1.964 (1.778–2.169)
24-h LC_90_ (95% confidence)	47.83 (37.83–71.41)	41.88 (37.37–49.09)	36.42 (29.16–49.60)	2.419 (2.126–2.887)	7.730 (7.006–8.955)	52.66 (49.13–57.71)	3.743 (3.273–4.472)
48-h LC_50_ (95% confidence)	15.59 (12.46–18.32)	26.60 (24.51–28.97)	6.773 (4.957–8.467)	1.304 (1.183–1.437)	4.979 (4.608–5.377)	36.91 (34.35–39.56)	1.533 (1.376–1.703)
48-h LC_90_ (95% confidence)	46.24 (40.60–55.31)	39.79 (35.89–46.73)	30.62 (24.39–42.18)	2.351 (2.060–2.822)	7.354 (6.659–8.469)	50.00 (46.18–55.42)	3.127 (2.714–3.773)
*Aedes albopictus*							
24-h LC_50_ (95% confidence)	N.T.	N.T.	34.20 (31.90–36.73)	N.T.	N.T.	N.T.	N.T.
24-h LC_90_ (95% confidence)	N.T.	N.T.	45.98 (42.28–51.38)	N.T.	N.T.	N.T.	N.T.
48-h LC_50_ (95% confidence)	N.T.	N.T.	31.13 (29.15–33.70)	N.T.	N.T.	N.T.	N.T.
48-h LC_90_ (95% confidence)	N.T.	N.T.	40.82 (37.09–47.24)	N.T.	N.T.	N.T.	N.T.
*Culex quinquefasciatus*							
24-h LC_50_ (95% confidence)	N.T.	N.T.	56.16 (51.10–61.54)	N.T.	N.T.	N.T.	N.T.
24-h LC_90_ (95% confidence)	N.T.	N.T.	104.7 (93.0–121.7)	N.T.	N.T.	N.T.	N.T.
48-h LC_50_ (95% confidence)	N.T.	N.T.	51.21 (46.52–56.27)	N.T.	N.T.	N.T.	N.T.
48-h LC_90_ (95% confidence)	N.T.	N.T.	97.91 (86.55–114.54)	N.T.	N.T.	N.T.	N.T.
**Mosquito Species**	***Piper* species**						**Permethrin**
	***P. montium***	***P. mutabile***	***P. nigrum***	***P. rubrum***	***P. rubrum***	***P. sarmentosum***	
	**Leaves & Stems**	**Leaves & Stems**	**Leaves & Stems**	**Leaves**	**Stems**	**Leaves & Stems**	
*Aedes aegypti*							
24-h LC_50_ (95% confidence)	1.925 (1.771–2.092)	1.850 (1.715–1.997)	8.805 (7.905–9.811)	24.98 (22.74–27.48)	30.87 (28.66–33.32)	22.82 (17.73–27.91)	6.4 × 10^−4^ (5.5 × 10^−4^–7.5 × 10^−4^)
24-h LC_90_ (95% confidence)	3.180 (2.784–3.793)	2.696 (2.442–3.108)	18.83 (16.15–23.13)	43.70 (38.35–52.39)	45.54 (40.14–54.01)	130.6 (88.7–256.8)	2.5 × 10^−3^ (1.9 × 10^−3^–3.44 × 10^−3^)
48-h LC_50_ (95% confidence)	1.691 (1.531–1.867)	1.695 (1.544–1.842)	6.909 (6.149–7.720)	22.15 (20.19–24.25)	26.09 (23.83–28.63)	18.42 (12.97–23.48)	N.T.
48-h LC_90_ (95% confidence)	3.024 (2.714–3.509)	2.641 (2.367–3.122)	15.49 (13.28–19.03)	37.18 (32.97–43.97)	44.80 (40.63–51.40)	126.8 (90.1–220.9)	N.T.
*Aedes albopictus*							
24-h LC_50_ (95% confidence)	N.T.	N.T.	43.92 (40.18–47.95)	N.T.	N.T.	49.24 (44.72–53.99)	2.3 × 10^−3^ (2.1 × 10^−3^–2.6 × 10^−3^)
24-h LC_90_ (95% confidence)	N.T.	N.T.	74.25 (66.15–86.73)	N.T.	N.T.	90.30 (80.19–105.31)	4.2 × 10^−3^ (3.8 × 10^−3^–4.9 × 10^−3^)
48-h LC_50_ (95% confidence)	N.T.	N.T.	39.19 (35.75–42.86)	N.T.	N.T.	38.33 (33.62–42.97)	N.T.
48-h LC_90_ (95% confidence)	N.T.	N.T.	67.14 (59.57–79.16)	N.T.	N.T.	86.32 (74.63–104.92)	N.T.
*Culex quinquefasciatus*							
24-h LC_50_ (95% confidence)	N.T.	N.T.	N.T.	N.T.	N.T.	N.T.	1.7 × 10^−2^ (1.5 × 10^−2^–1.8 × 10^−2^)
24-h LC_90_ (95% confidence)	N.T.	N.T.	N.T.	N.T.	N.T.	N.T.	2.9 × 10^−2^ (2.7 × 10^−2^–3.3 × 10^−2^)
48-h LC_50_ (95% confidence)	N.T.	N.T.	N.T.	N.T.	N.T.	N.T.	N.T.
48-h LC_90_ (95% confidence)	N.T.	N.T.	N.T.	N.T.	N.T.	N.T.	N.T.

N.T. = Not tested.

## Supplementary Materials

The following are available, Figures S1–S16: Total Ion Chromatograms of Essential Oils of *Piper* Species from Central Vietnam.
