# Supplementary material for: Chemical Compositions and Mosquito Larvicidal Activities of Essential Oils from Piper Species Growing Wild in Central Vietnam
Source: Molecules. 2019 Oct 27;24(21):3871. doi: 10.3390/molecules24213871 (PMC6864731; doi:10.3390/molecules24213871)
Supplement: Supplementary file 1 [file molecules-24-03871-s001.pdf]

Chemical Compositions and Mosquito Larvicidal Activities of Essential Oils from *Piper* Species Growing Wild in Central Vietnam

Lê Thị Hương [1], Nguyễn Huy Hùng [2,*], Đỗ Ngọc Đài [3], Thiều Anh Tài [4], Vũ Thị Hiền [5], Prabodh Satyal [6], and William N. Setzer [6,7,*]

Supplementary Material

Total Ion Chromatograms of Essential Oils of *Piper* Species from Central Vietnam.

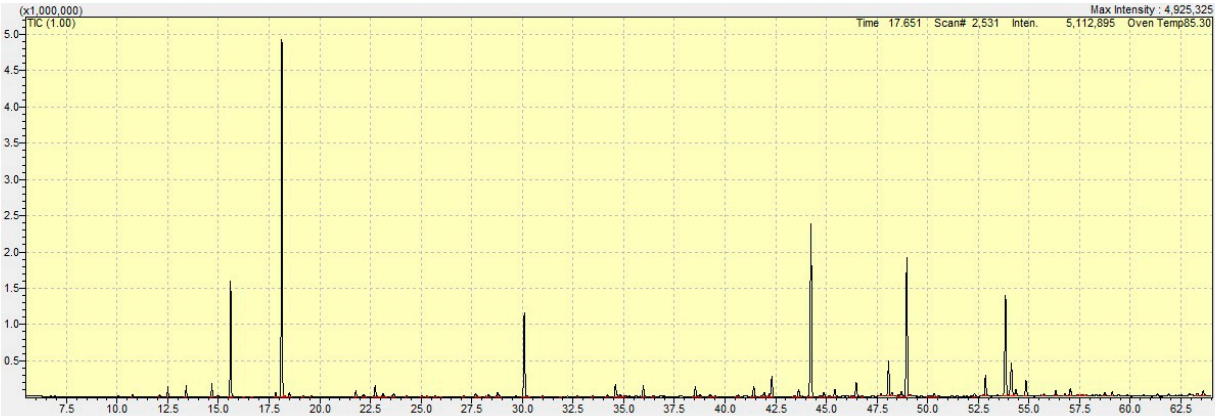

Figure S1. Total ion chromatogram of *Piper arboricola* leaves and stems essential oil.

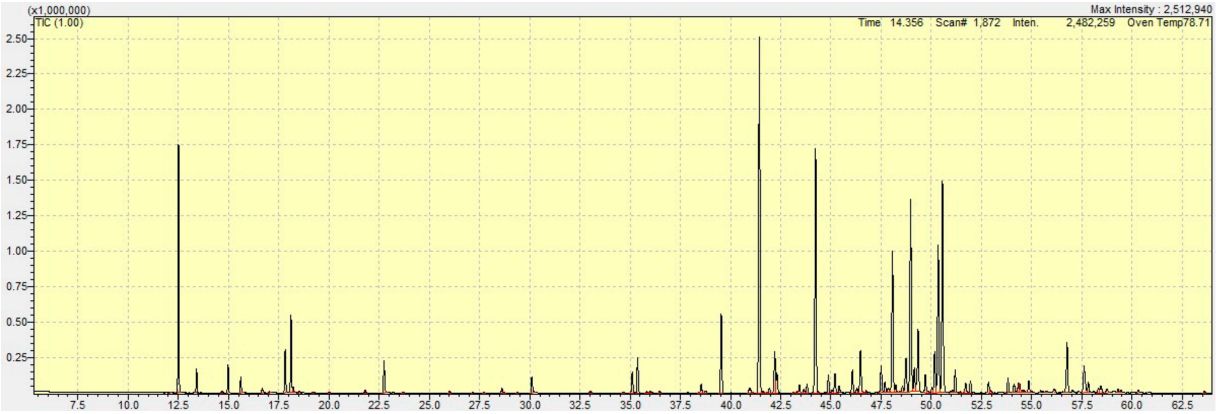

Figure S2. Total ion chromatogram of *Piper arboricola* leaf essential oil.

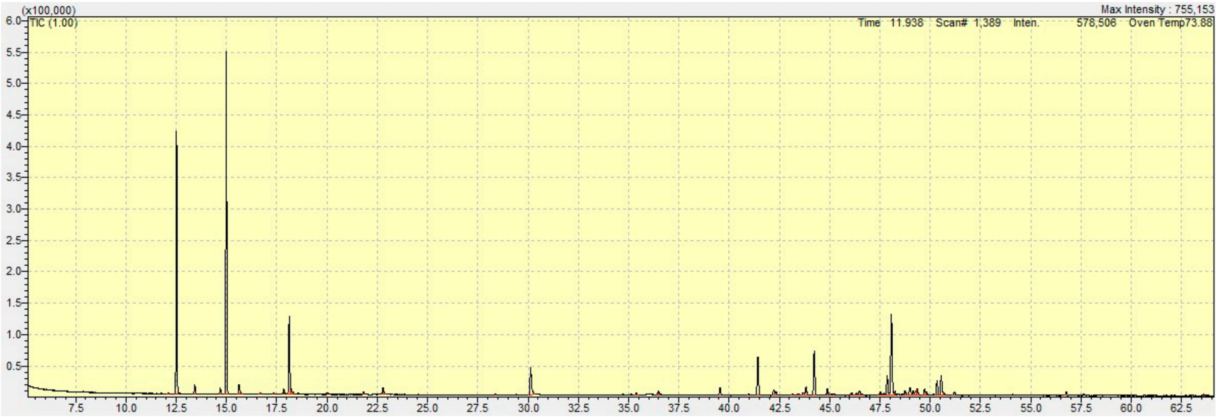

Figure S3. Total ion chromatogram of *Piper arboricola* stem essential oil.

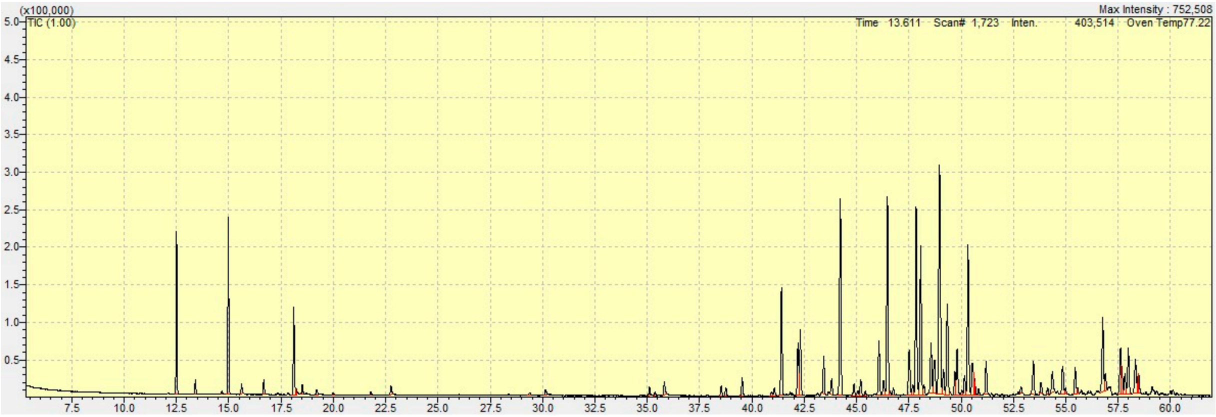

Figure S4. Total ion chromatogram of *Piper bavinum* leaves and stems essential oil.

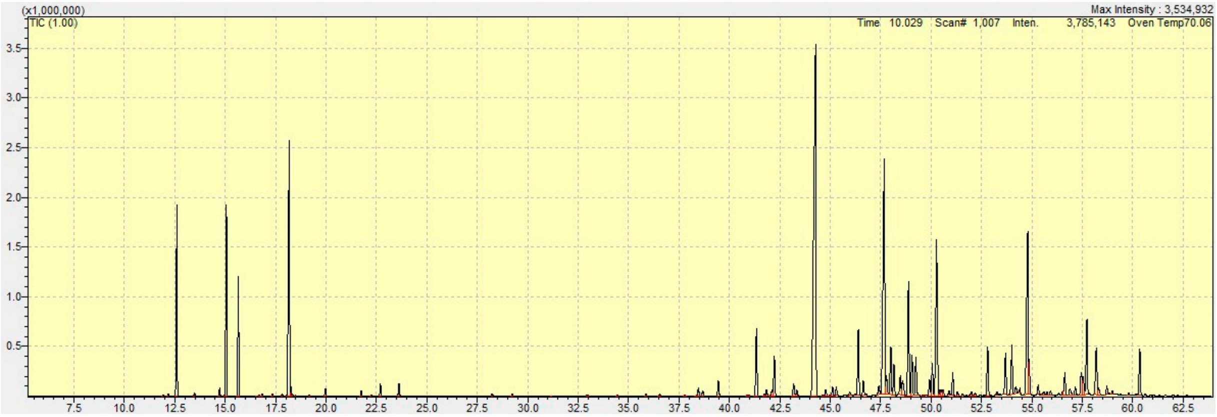

Figure S5. Total ion chromatogram of *Piper cambodianum* leaves and stems essential oil.

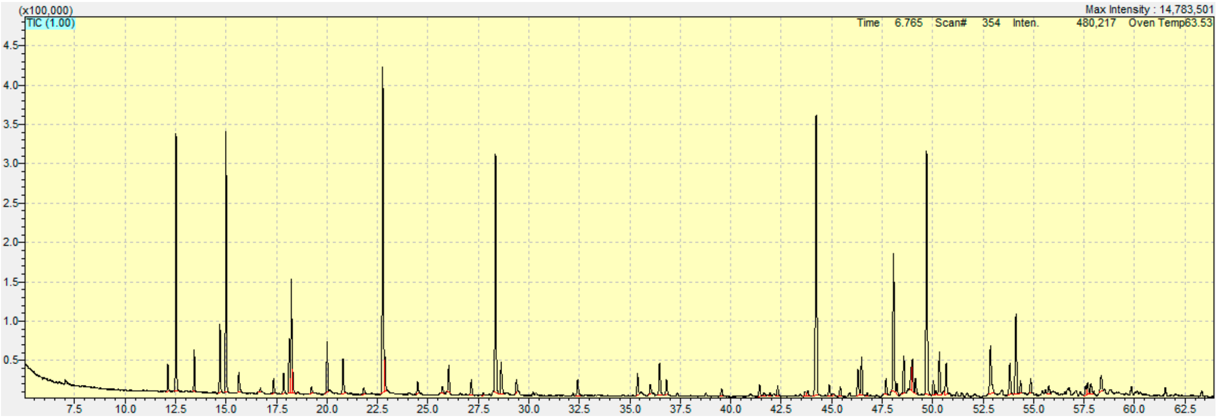

Figure S6. Total ion chromatogram of *Piper caninum* leaves and stems essential oil.

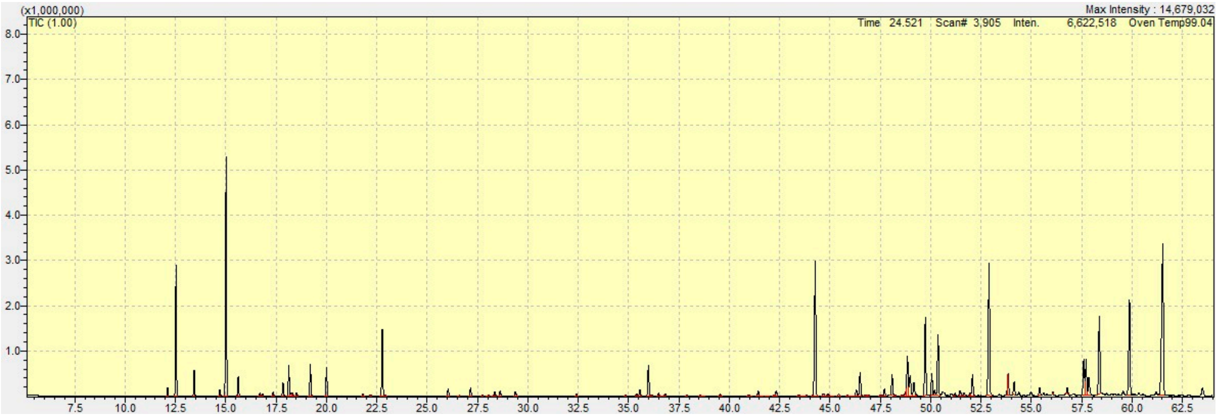

Figure S7. Total ion chromatogram of *Piper longum* leaves and stems essential oil.

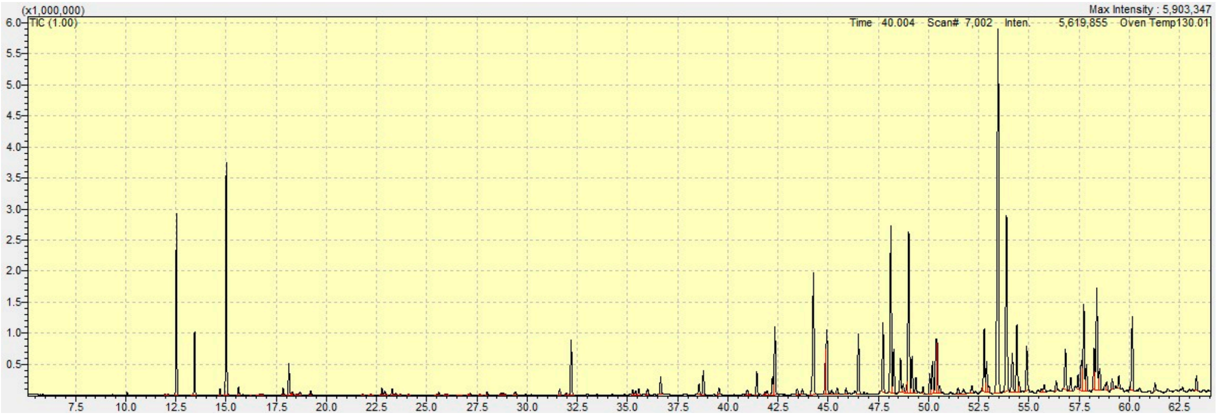

Figure S8. Total ion chromatogram of *Piper mekongense* leaves and stems essential oil.

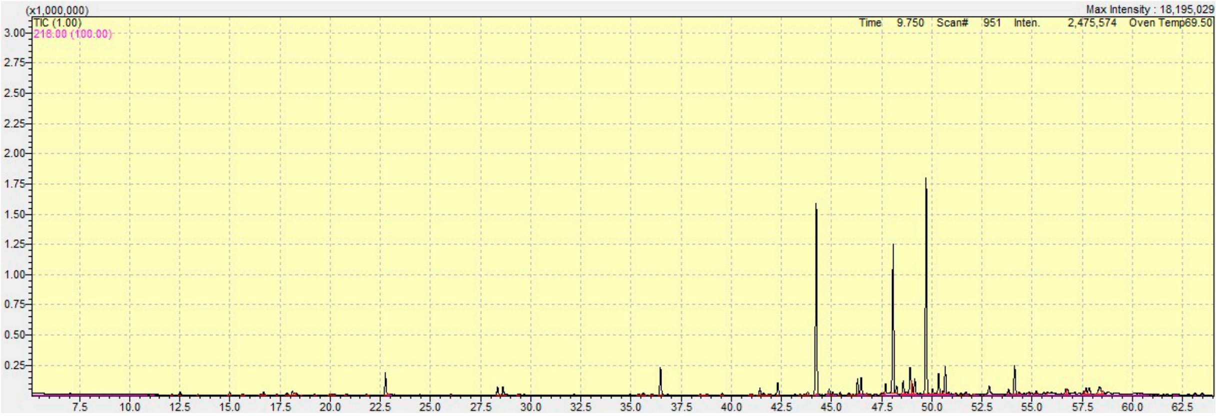

Figure S9. Total ion chromatogram of *Piper montium* leaves and stems essential oil.

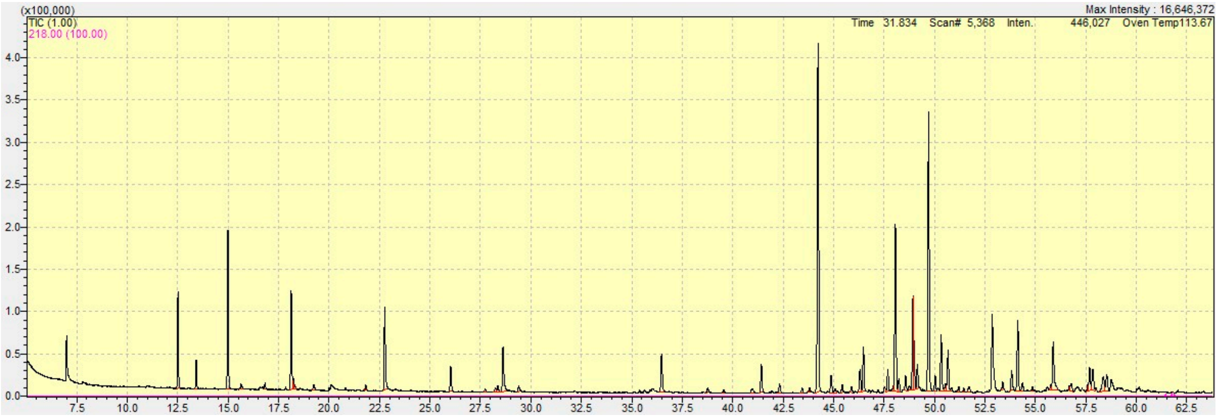

Figure S10. Total ion chromatogram of *Piper mutabile* leaves and stems essential oil.

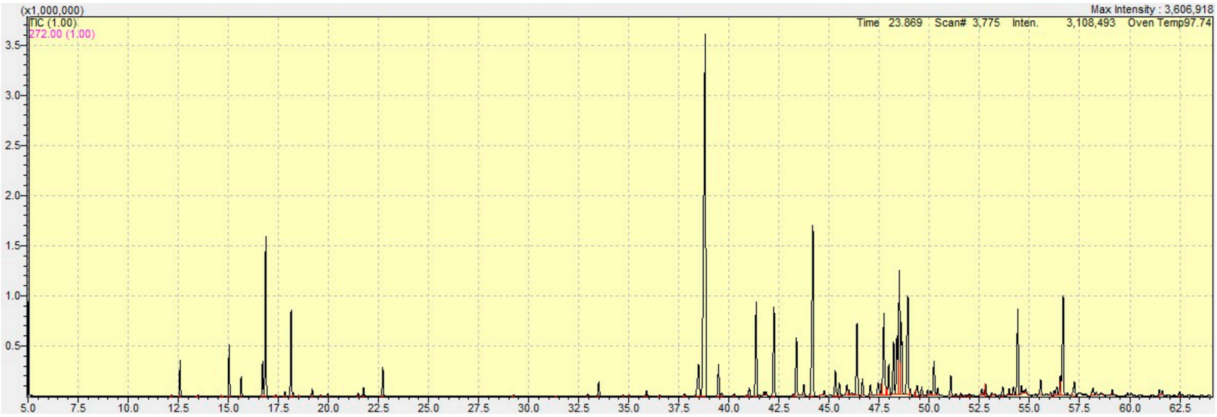

Figure S11. Total ion chromatogram of *Piper nigrum* leaves and stems essential oil.

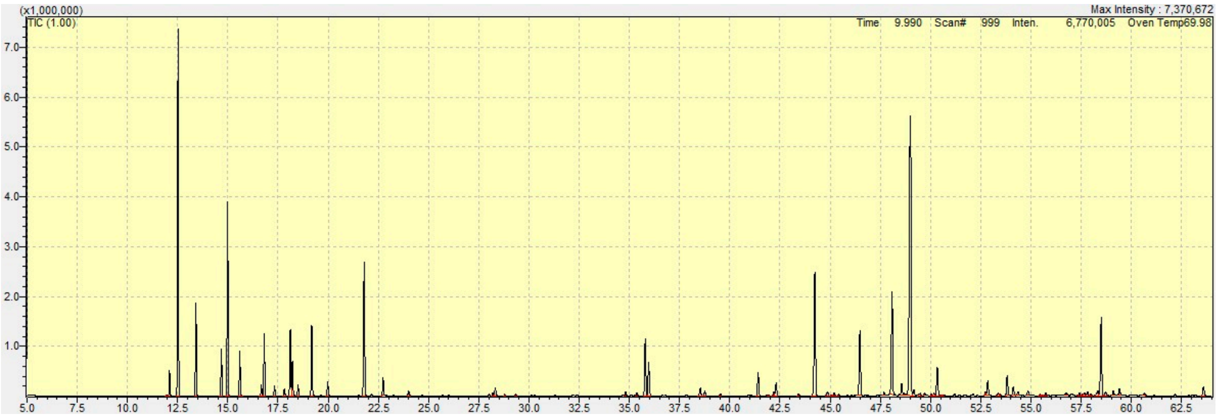

Figure S12. Total ion chromatogram of *Piper politifolium* leaves and stems essential oil.

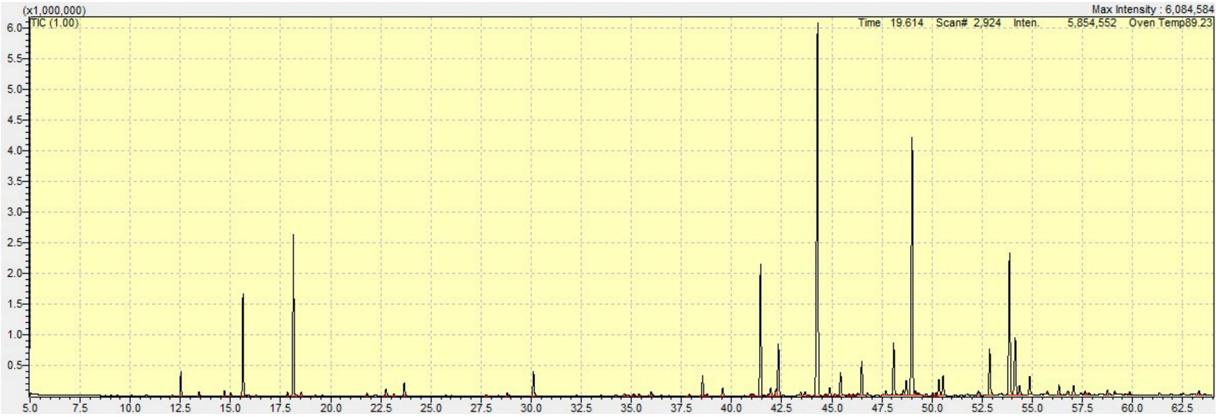

Figure S13. Total ion chromatogram of *Piper rubrum* leaf essential oil.

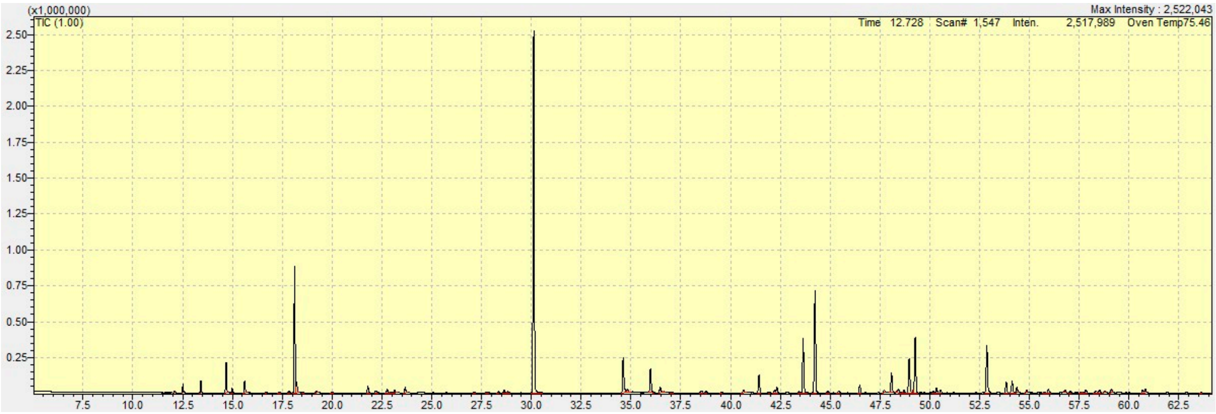

Figure S14. Total ion chromatogram of *Piper rubrum* stem essential oil.

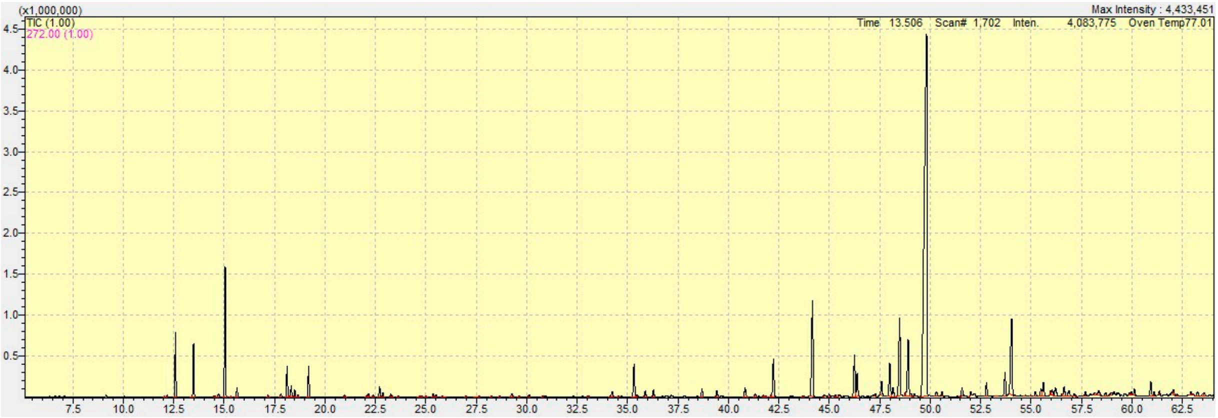

Figure S15. Total ion chromatogram of *Piper sarmentosum* leaves and stems essential oil.

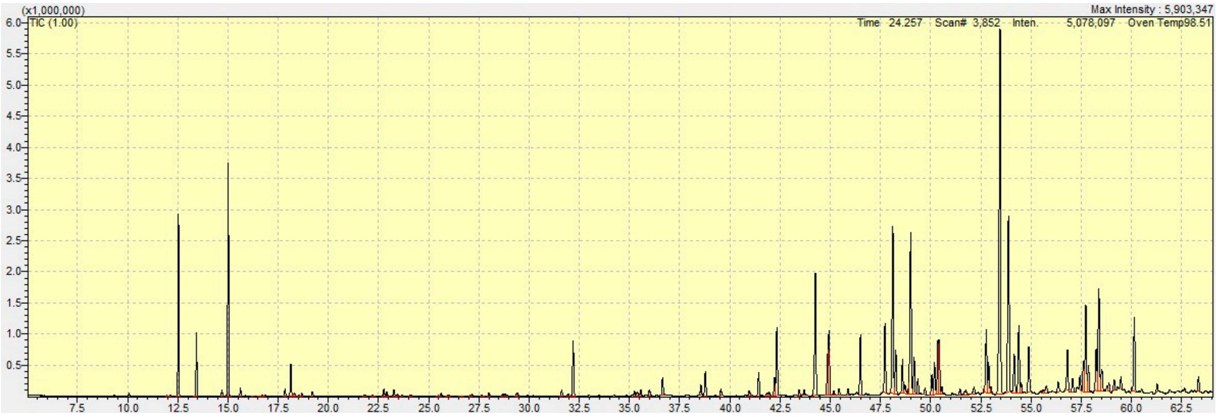

Figure S16. Total ion chromatogram of *Piper umbellatum* leaves and stems essential oil.
